# Bioinformatic Analysis of *IKK* Complex Genes Expression in Selected Gastrointestinal Cancers

**DOI:** 10.3390/ijms25189868

**Published:** 2024-09-12

**Authors:** Marta Żebrowska-Nawrocka, Dagmara Szmajda-Krygier, Adrian Krygier, Agnieszka Jeleń, Ewa Balcerczak

**Affiliations:** 1 Department of Pharmaceutical Biochemistry and Molecular Diagnostics, Medical University of Lodz, Muszynskiego 1, 90-151 Lodz, Poland; dagmara.szmajda@umed.lodz.pl (D.S.-K.); adrian.krygier@umed.lodz.pl (A.K.); agnieszka.jelen@umed.lodz.pl (A.J.); ewa.balcerczak@umed.lodz.pl (E.B.); 2 Laboratory of Molecular Diagnostics, Brain Laboratories, Medical University of Lodz, Czechoslowacka 4, 92-216 Lodz, Poland

**Keywords:** *CHUK*, *IKBKB*, *IKBKG*, gastrointestinal cancer, expression, prognostic marker

## Abstract

Gastrointestinal cancers account for over a quarter of all cancer cases and are associated with poor prognosis and high mortality rates. The IKK complex (the canonical I kappa B kinase), comprising the *CHUK*, *IKBKB*, and *IKBKG* genes, plays a crucial role in activating the NF-kB signaling pathway. This study aimed to analyze publicly available bioinformatics data to elucidate the oncogenic role of *IKK* genes in selected gastrointestinal cancers. Our findings reveal that *IKBKB* and *IKBKG* are significantly upregulated in all examined cancers, while *CHUK* is upregulated in esophageal carcinoma and stomach adenocarcinoma. Additionally, the expression of *IKK* genes varies with histological grade and nodal metastases. For instance, in stomach adenocarcinoma, *CHUK* and *IKBKB* are upregulated in higher histological grades and greater lymph node infiltration. Lower expression levels of *CHUK*, *IKBKB*, and *IKBKG* in stomach adenocarcinoma and *IKBKB* in esophageal squamous cell carcinoma correlate with shorter overall survival. Conversely, in esophageal adenocarcinoma, reduced *IKBKG* expression is linked to longer overall survival, while higher *IKBKB* expression in colon adenocarcinoma is associated with longer overall survival. Given the significant role of *IKK* genes in the development and progression of selected gastrointestinal cancers, they hold potential as prognostic markers and therapeutic targets, offering valuable insights for clinical practice.

## 1. Introduction

Gastrointestinal cancers (GI) are a heterogeneous group of diseases that represent more than one-quarter of cancers globally and more than one-third of all malignancies. It is estimated that 4.8 million new cases were reported in 2018, with a mortality rate of 3.4 million people, worldwide. According to the current dynamics of change, it is projected that morbidity and mortality due to GI will double over the next 20 years. Gastrointestinal (GI) cancers share common risk factors but differ significantly in etiology and epidemiology. Over half of GI cancers are attributed to modifiable factors, such as alcohol consumption, smoking, infections, diet, and obesity [1,2]. Among GI cancers, esophageal carcinoma (ESCA), stomach adenocarcinoma (STAD), and colorectal cancers (CRC)—including colon adenocarcinoma (COAD) and rectum adenocarcinoma (READ)—are the most prevalent. ESCA, divided histologically into squamous cell carcinoma (SCC) and adenocarcinoma (ECA), is one of the most aggressive malignancies with poor survival rates. Its primary risk factors include poor diet, smoking, excessive alcohol consumption, and HPV infection [2,3,4]. COAD is the third most common and second deadliest cancer globally. Its etiology involves both environmental and genetic factors, with the pathological stage at diagnosis being the most crucial prognostic indicator. Since 2004, COAD incidence has declined by 3% annually, likely due to increased cancer screening [5,6,7]. Rectum adenocarcinoma shares similar diagnosis and mortality rates with colon adenocarcinoma but has distinct environmental and genetic risk factors. The progression from normal rectal epithelium to invasive cancer typically spans about 10 years, involving environmental, genetic, somatic, and germline factors. Pathological stage at diagnosis remains the primary prognostic indicator for READ [8,9]. STAD, the fourth most common digestive tract cancer, is highly aggressive and heterogeneous. Both environmental and genetic factors influence its development, with incidence rising with age. According to Lauren’s criteria, STAD includes enteric and diffuse subtypes, each with distinct clinical and morphological characteristics. Incidence has declined due to improved hygiene, food preservation, fresh fruit and vegetable consumption, and *Helicobacter pylori* eradication. Despite this, gastric cancer continues to pose a significant health challenge with poor prognosis [10,11].

Several susceptibility genes, such as *MHL1*, *MSH2*, *MSH6*, *PMS2*, and *EPCAM* (linked to Lynch syndrome), are associated with GI malignancies. These genes, mainly linked to CRC, also relate to gastric and pancreaticobiliary cancers. However, few GI cancers can be directly tied to known cancer-associated genes, which does not fully explain the global rise in GI cancer incidence. Early detection and treatment initiation are vital for improving prognosis. Despite advances in medicine, surgery remains the predominant treatment for GI cancers. Genetic factors are crucial in carcinogenesis and may guide prognosis assessment and therapy selection [12,13].

The presented study is focused on the crucial NF-κB signaling pathway (nuclear factor kappa-light-chain-enhancer of activated B cells signaling pathway), especially the IKK complex, which plays an important role in its regulation.

The multimeric canonical I kappa B kinase (IKK) complex is a main regulator of the NF-κB signaling pathway, and it is composed of the two catalytic kinases subunits: CHUK (IKBKA, IKK1 or IKKα) and IKBKB (IKK2 or IKKβ), and a regulatory non-catalytic subunit IKBKG (NEMO—NF-κB essential modulator, IKK3 or IKKγ). Through phosphorylation, ubiquitination, and degradation of the IkB inhibitor, this complex leads to the release of NF-κB, which functions as a transcription factor for many genes in the cell nucleus. Activation of this pathway and related genes is associated with proliferation, differentiation, protection of the cell against apoptosis, and immune response. Both CHUK and IKBKB subunits have a homologous structure and a NEMO-binding domain. IKBKG has in its structure, among others, a zinc finger domain and a leucine zipper. It is responsible for binding the remaining kinase subunits and activating them. It is indicated that the presence of three subunits is important in the canonical activation of the pathway, but mainly IKBKB and IKBKG. CHUK is found as a homodimer in the non-canonical NF-κB pathway [14,15,16,17,18,19].

Activation of the NF-κB pathway itself and the IKK kinase is identified in many diseases, including cancer. Studies on mice with *IKBKG* deletion in pancreatic cells showed that the lack of expression of this gene was important in limiting the ability to form metastases and had a positive effect on the length of survival [20]. Other studies in a mouse model have shown that the presence of *CHUK* was important in tumor initiation and proliferation in colon cancer [21]. The role of *CHUK* in carcinogenesis may also be confirmed by studies in which *CHUK* activation was associated with increased promotion of metastasis in prostate cancer [22]. In another study on cell lines, CHUK via phosphorylation activated the stabilizer of c-MYC, which resulted in the upgraded proliferation and inhibited apoptosis of cells and promoted cancerogenesis [23].

Similar to other cancers, also in those affecting the gastrointestinal tract, the NF-κB pathway may be activated by various elements, such as growth factors, stress, cytokines, but also viral or bacterial products. In vitro studies have shown that *Helicobacter pylori* infection activates *CHUK* and *IKBKB* in the gastric mucosa, which is associated with the inflammatory process and carcinogenesis [24]. The study on an animal model showed that the reduction of the *CHUK* gene was associated with chronic fungal infection and the subsequent development of esophageal squamous cell carcinoma [25].

It is worth noting that the IKK complex may also influence cancer development independently of the NF-κB pathway. For example, in vitro studies on colorectal cancer cell lines showed that the mutated *BRAF* gene could activate *CHUK* and then, independently of NF-κB, led to the activation of genes identified as their common targets, which were involved in carcinogenesis promoting [26]. Another study on colorectal cancer cell lines demonstrated the activating effect of *CHUK*, independently of NF-κB, on SMRT (silencing mediator of retinoic acid and thyroid hormone receptor) phosphorylation and activation of genes from the NOTCH pathway, also promoting the proliferation of cancer cells [27]. 

As the genes included in the IKK complex have such a strong influence on many processes determining autonomy as well as escape from immune control, the present study subjected them to bioinformatic analysis to determine their importance in the pathogenesis of neoplastic changes in the GI tract. To the best of our knowledge, this is the first such comprehensive work to assess the role of all *IKK* complex genes in selected gastrointestinal cancers.

## 2. Results

### 2.1. The Pan-Cancer Analysis of the mRNA Level for the IKK Genes Complex

**Figure 1:**
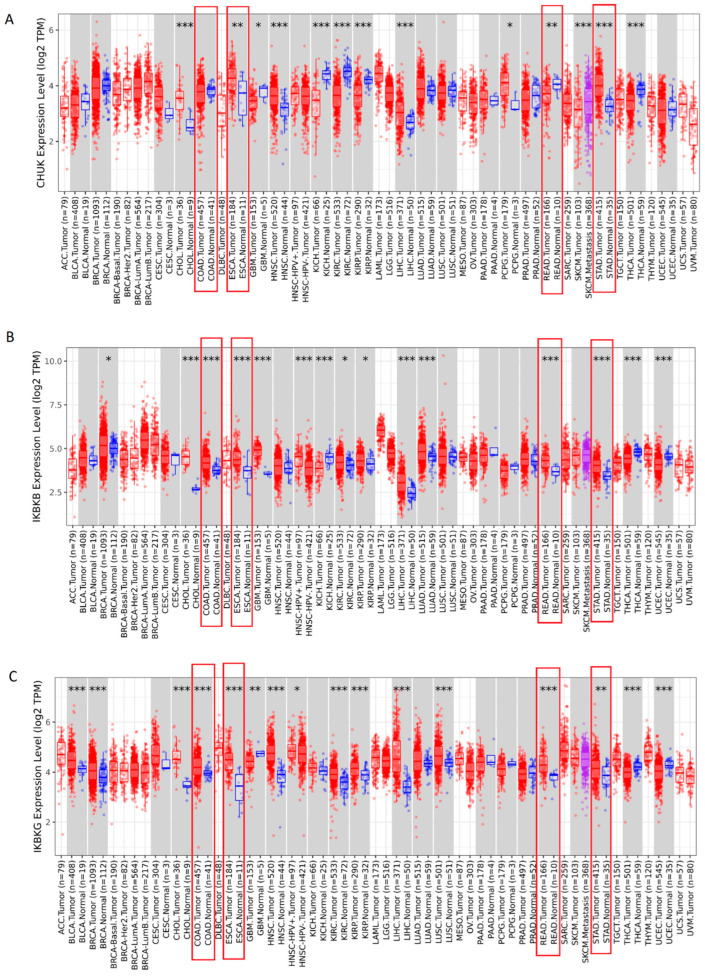
The expression levels of (**A**) the *CHUK* gene, (**B**) the *IKBKB* gene, and (**C**) the *IKBKG* gene in pan-cancer using TIMER2.0, which showed expression in TCGA cancers (red box), the corresponding normal tissues (blue box) and metastasis tissue (purple box)wosuoyi; * *p* < 0.05; ** *p* < 0.01; *** *p* < 0.001 (Data for GI cancers are marked with red frames).

The first analysis compared the expression of the *IKK* complex genes with those collected in the TIMER2 database of various cancer types and normal tissues. As shown in Figure 1A–C, the expression of all IKK complex genes depends on the type of cancer. In most cancers, these genes are overexpressed in tumors compared to normal tissue. The levels of *IKBKB* (*p* < 0.001) and *IKBKG* mRNA (*p* < 0.01) were higher in COAD, ESCA, READ, and STAD in comparison with normal tissue (Figure 1B,C). The *CHUK* gene was overexpressed in ESCA (*p* = 0.0055) and STAD (*p* < 0.001) and underexpressed in READ (*p* = 0.0067) compared to normal tissue (Figure 1A). 

**Figure 2:**
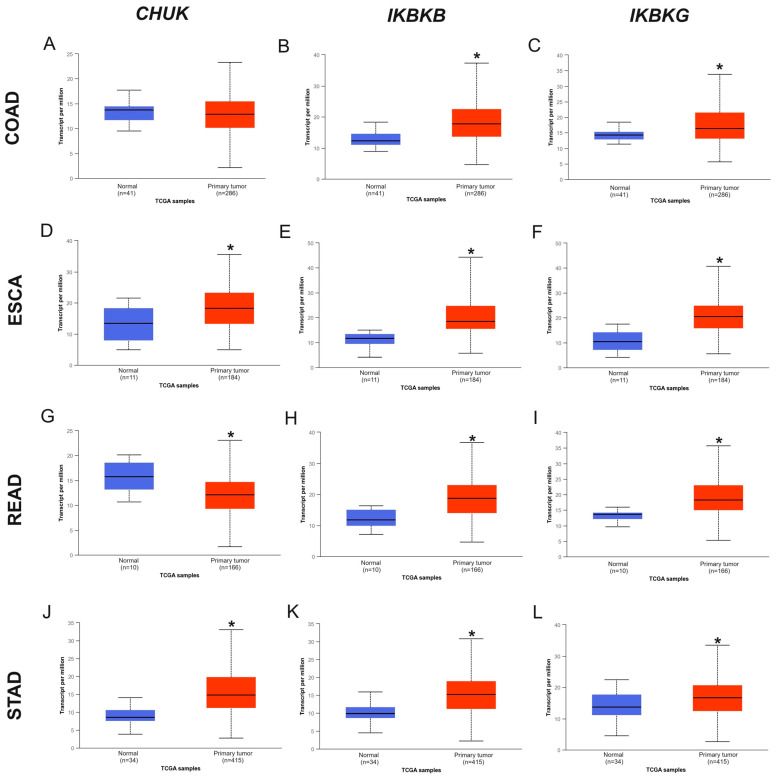
Expression profiling of IKK complex genes in GI cancers and normal tissue, from UALCAN: (**A**) *CHUK* gene; (**B**) *IKBKB* gene; (**C**) *IKBKG* gene in COAD; (**D**) *CHUK* gene (**E**) *IKBKB* gene; (**F**) *IKBKG* gene in ESCA; (**G**) *CHUK* gene; (**H**) *IKBKB* gene; (**I**) *IKBKG* gene in READ; (**J**) *CHUK* gene; (**K**) *IKBKB* gene; (**L**) *IKBKG* gene in STAD (tumor—red box, normal—blue box; * *p* < 0.05).

**Figure 3:**
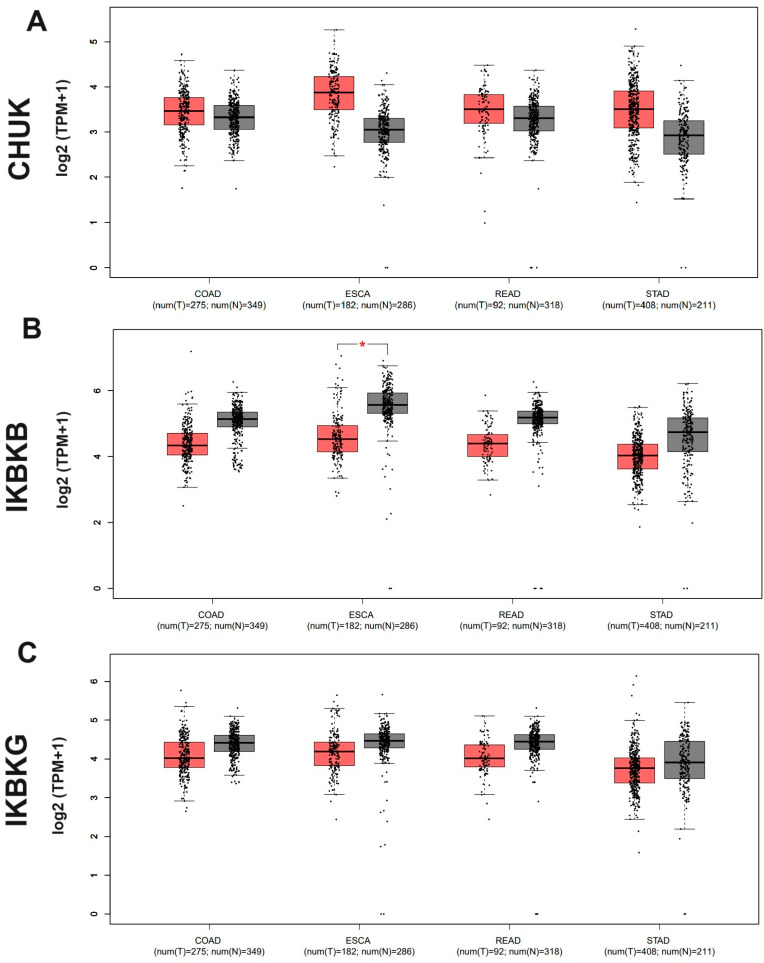
The expression level of (**A**) the *CHUK* gene in COAD, ESCA, READ, and STAD; (**B**) the *IKBKB* gene in COAD, ESCA, REDA, and STAD; and (**C**) the *IKBKG* gene in COAD, ESCA, READ, and STAD and corresponding normal tissue, from GEPIA (tumor—red box, normal—gray box; * *p* < 0.01).

Additionally, IKK complex gene expression was compared using UALCAN and GEPIA tools. The results obtained from the UALCAN database were similar to those from TIMER2 (Figure 2). GEPIA analysis indicated lower expression of the *IKBKB* and *IKBKG* genes in all examined GI cancers compared to normal cells (Figure 3B,C); however, in most cancers, the difference was not statistically significant. The exception was the *IKBKB* gene in ESCA (*p* < 0.01, Figure 3B). Differences between the results of the databases may result from the fact that the data come from TCGA in TIMER and UALCAN and from TCGA and GTX in the GEPIA2 database, and there may be differences in group sizes and the use of different statistical tests.

### 2.2. The Association between IKK Complex Genes mRNA Levels and GI Cancer Histological Type or Subtype

In the next step, the expression of the studied genes was analyzed depending on the histological types or subtypes of COAD, ESCA, READ, and STAD. In ESCA, the CHUK gene showed significantly higher expression in the case of adenocarcinoma compared to squamous cell carcinoma (*p* < 0.001). Conversely, for the IKBKG gene, higher expression was noted for the squamous cell carcinoma type of ESCA (*p* < 0.001). CHUK gene expression was significantly higher in the group of patients with the tubular-intestinal-adenocarcinoma subtype than in the subgroup with diffuse adenocarcinoma of STAD cancer (*p* = 0.0103). No other significant relationships were found regarding the connection between CHUK, IKBKB, or IKBKG gene expression and cancer type. All data are summarized in Table 1.

### 2.3. The Correlations between the mRNA Level of the IKK Complex Genes and Clinicopathological Features

**Figure 4:**
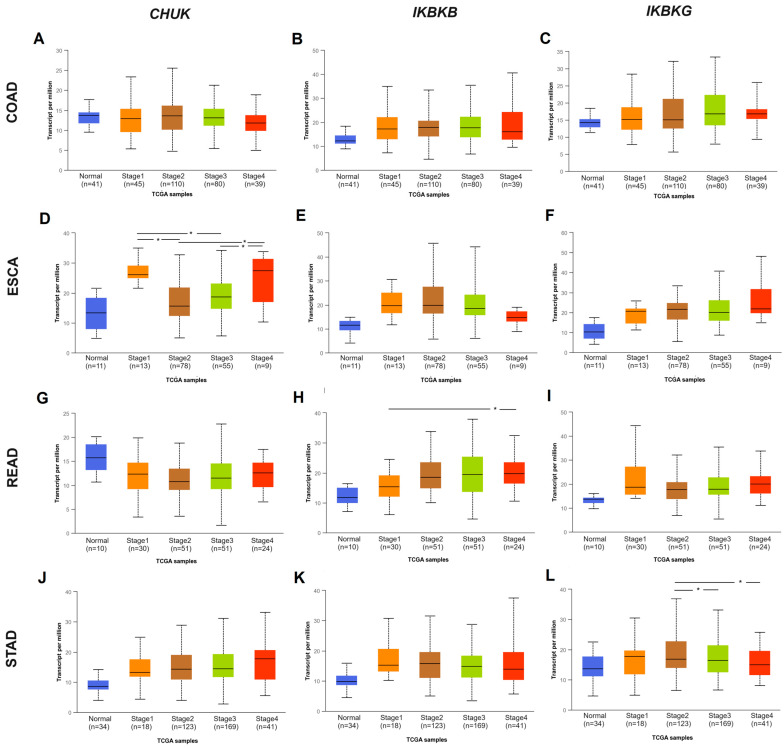
Differences in IKK complex genes expression according to individual clinical cancer stage: (**A**) *CHUK* gene; (**B**) *IKBKB* gene; (**C**) *IKBKG* gene in COAD; (**D**) *CHUK* gene; (**E**) *IKBKB* gene; (**F**) *IKBKG* gene in ESCA; (**G**) *CHUK* gene; (**H**) *IKBKB* gene; (**I**) *IKBKG* gene in READ; (**J**) *CHUK* gene; (**K**) *IKBKB* gene; (**L**) *IKBKG* gene in STAD based on UALCAN web tool (* *p* < 0.05). Accessed 17–19 January 2023.

The next stage examined the association of gene expression level with clinical stage, metastases to local lymph nodes, and histological grade in the investigated GI cancers. In ESCA, *CHUK* gene expression was higher in stages 1 and 4 than in stages 2 (*p* = 0.0014; 0.0214, respectively) or 3 (*p* = 0.0028; 0.0354, respectively). This could be connected with the different sizes of the subgroups used in the analysis. In READ, only *IKBKB* gene expression was differentiated between cancer stages (Figure 4). *IKBKG* gene expression was correlated only with STAD staging, being upregulated in stage 2 compared to stage 3 (*p* = 0.0293) or to stage 4 (*p* = 0.0485) (Figure 4).

**Figure 5:**
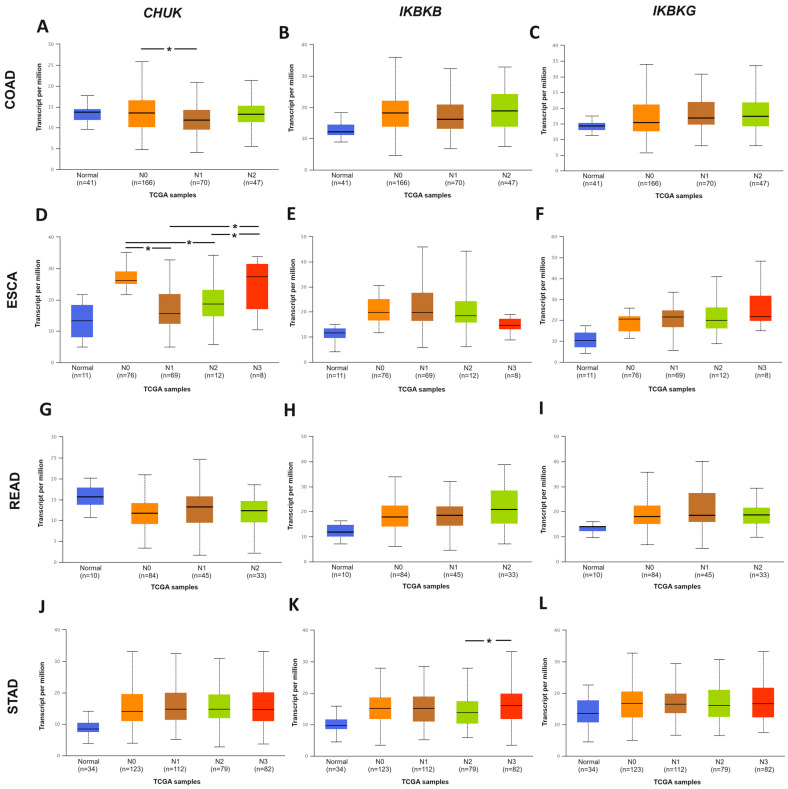
Differences in IKK complex genes expression according to nodal metastasis: (**A**) *CHUK* gene; (**B**) *IKBKB* gene; (**C**) *IKBKG* gene in COAD; (**D**) *CHUK* gene; (**E**) *IKBKB* gene; (**F**) *IKBKG* gene in ESCA; (**G**) *CHUK* gene; (**H**) *IKBKB* gene; (**I**) *IKBKG* gene in READ; (**J**) *CHUK* gene; (**K**) *IKBKB* gene; (**L**) *IKBKG* gene in STAD based on UALCAN web tool (* *p* < 0.05; N0—metastases into regional lymph node; N1—metastases in one to three axillary lymph nodes; N2—metastases in four to nine axillary lymph nodes; N3—metastases in ten or more axillary lymph nodes). Accessed 17–18 January 2023.

In the next step, the invasion to local lymph nodes was determined. It was found that neither the occurrence of lymph node metastases nor their number correlated with the level of IKBKG gene expression. In contrast, the IKBKB gene was upregulated in the subgroup of patients determined as N3 compared to N2 (*p* = 0.0363) for STAD cancer. For CHUK, the gene expression was significantly lower in N1 than in N0 in COAD patients. Interestingly, the level of CHUK gene expression was upregulated in NO compared to N1 (*p* = 0.0014) or to N2 (*p* = 0.0028) for ESCA; in addition, this gene demonstrated higher expression in N3, a more advanced stage of nodal metastases, compared to N1 (*p* = 0.0214) and N2 (*p* = 0.0354) (Figure 5).

**Figure 6:**
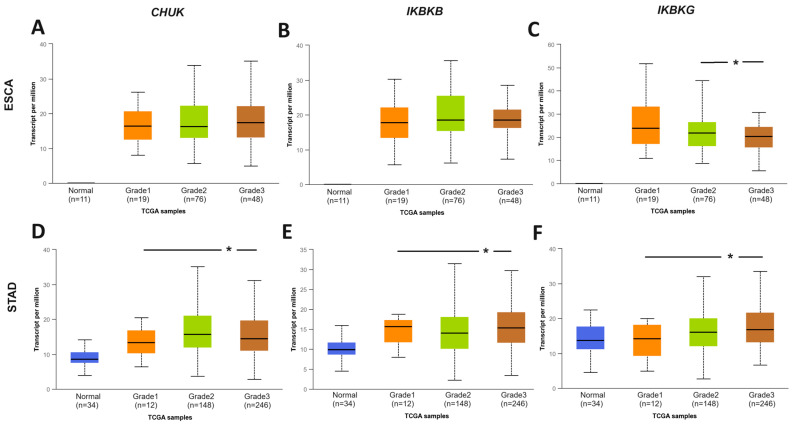
Differences in IKK complex genes expression according to histological grade: (**A**) *CHUK* gene, (**B**) *IKBKB* gene, (**C**) *IKBKG* gene in COAD; (**D**) *CHUK* gene, (**E**) *IKBKB* gene (**F**) *IKBKG* gene in STAD based on UALCAN web tool (* *p* < 0.05; Grade 1—well differentiated (low grade); Grade 2—moderately differentiated (intermediate grade); Grade 3—poorly differentiated (high grade). UALCAN, Access 17–18 January 2023.

For histological grade, only data on ESCA and STAD patients were available in the UALCAN database. In ESCA, the IKBKG mRNA level was significantly higher in Grade 2 than in Grade 3 (*p* < 0.001). In STAD, CHUK and IKBKG genes were upregulated in Grade 3 compared to Grade 1 (*p* < 0.001), while IKBKB gene expression was significantly lower in Grade 3 than in Grade 1 (*p* < 0.001) (Figure 6).

### 2.4. The Dependences between mRNA Level and Certain Cancer-Related Risk Factors

#### 2.4.1. Weight

In the case of abnormal body weight, the expression of all three investigated genes only correlated with the ESCA subtype. *IKBKB* and *IKBKG* genes were upregulated in patients with normal weight compared to those who were of extreme weight (*p* = 0.0287; 0.0083, respectively) or extreme obesity (*p* = 0.0006 for *IKBKG*). The *IKBKG* was downregulated in the subgroup of patients: extreme weight and obese than normal weight (*p* = 0.0083; *p* = 0.0009, respectively). In contrast, *CHUK* expression increased with growing weight (*p* = 0.0413; 0.0025; 0.0388, respectively) (Supplementary Materials—Figure S1).

#### 2.4.2. Smoking and Alcohol Consumption

In ESCA, *IKBKG* gene expression was significantly higher in active smokers than those that had not smoked for less than 15 years (*p* = 0.0105). The mRNA levels of all analyzed genes in ESCA were also correlated with the frequency of alcohol consumption per week. *CHUK* expression was upregulated in patients who drank 7 days per week compared to patients who drank 4 or 5 days per week (*p* < 0.001). *IKBKB* gene expression was lower in alcohol drinkers compared to non-drinkers. However, expression increased with the number of days per week that patients consumed alcohol. Regarding *IKBKG*, higher expression levels were observed in patients who drank alcohol 2 or 7 days per week compared to non-drinkers (Supplementary Materials—Figure S2). 

#### 2.4.3. Helicobacter Pylori Infection

The information about the *Helicobacter pylori* infection status and *IKK* gene expression in the UALCAN database was only available for patients with STAD. The expression of the *CHUK* gene was lower in patients infected with *H. pylori* than in uninfected patients (*p* = 0.0089). Lower *IKBKB* gene expression was also noted among patients with *H. pylori* infection, but this was not statistically significant. *IKBKG* gene expression was not correlated with infection (Supplementary Materials—Figure S2).

### 2.5. The Correlation between the mRNA Level of IKK Complex Members and Patient Overall Survival in COAD, ESCA, READ, and STAD

The correlation between *CHUK*, *IKBKB*, *IKBKG*, and the overall survival (OS) of patients with COAD, ESCA, READ, and STAD cancer was assessed using the KM plotter database. In COAD patients, only *IKBKB* gene expression was associated with survival time; higher *IKBKB* expression was connected with a longer OS of patients (*p* = 0.039). No significant correlation between OS time and gene expression was demonstrated for READ.

**Figure 7:**
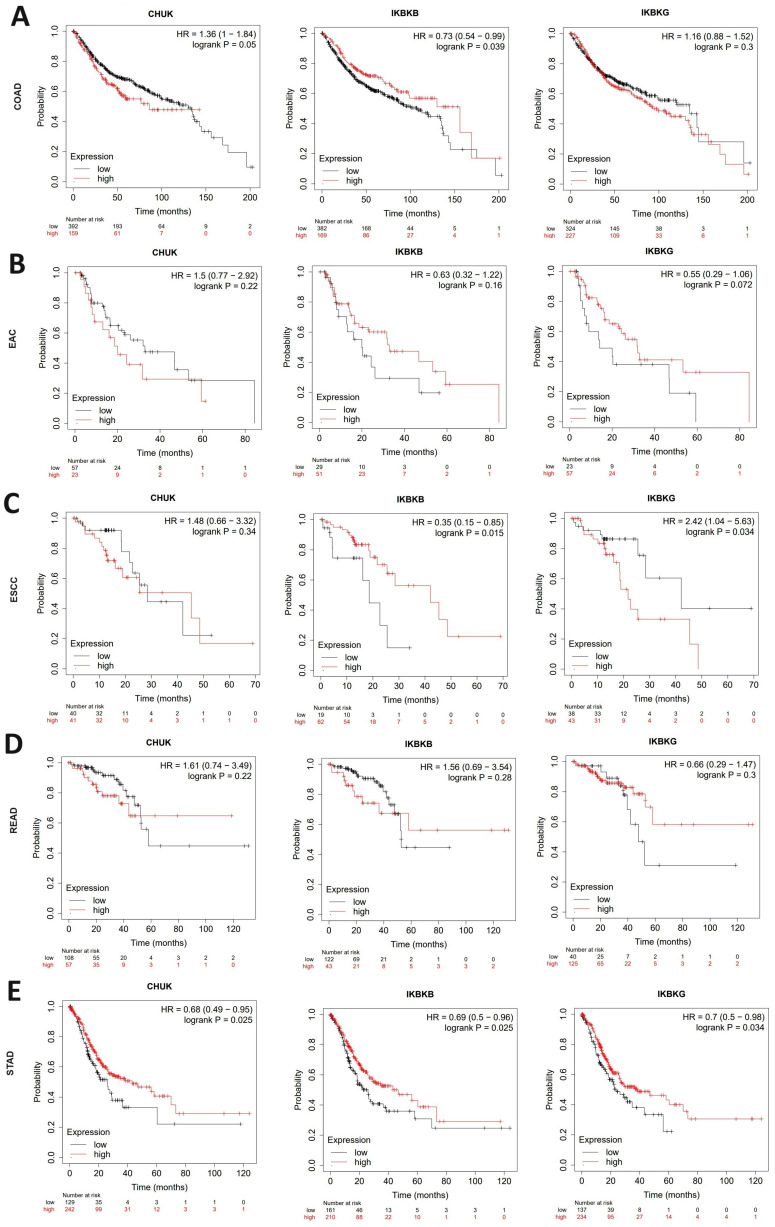
The correlation of IKK complex genes expression with patient overall survival in (**A**) COAD, (**B**) EAC, (**C**) ESCA, (**D**) READ, and (**E**) STAD based on the Kaplan-Meier plotter (access: 4–5 September 2023).

In the case of ESCA, KM plotter database analysis was possible for esophageal adenocarcinoma (EAC) and esophageal squamous cell carcinoma (ESCC) patients. Downregulated *IKBKB* gene expression was correlated with shorter OS (*p* = 0.015) in the esophageal squamous cell carcinoma subtype of ESCA. For the *IKBKG* gene in the ESCC subgroup, low gene expression was found to have a positive effect on overall survival (*p* = 0.034). In the STAD, the downregulation of *CHUK*, *IKBKB*, and *IKBKG* mRNA levels were correlated with shorter OS (*p* = 0.025; 0.025; 0.034, respectively). See Figure 7.

### 2.6. Association of IKK Genes Expression and Immune Cell Infiltration in COAD, ESCA, READ, and STAD

The interactions between the expression of the studied genes and immune cell infiltration in the tumor microenvironment were assessed in COAD, ESCA, READ, and STAD. The results of the assessment, together with exact *p*-values, are given in Supplementary Materials—Figures S3–S5. The *CHUK* gene mRNA level was positively correlated with tumor purity (lack of infiltrating cells) for ESCA and STAD tumors, but significant connections were only noted in ESCA. It may indicate that *CHUK* expression is higher in cancer cells than the cells of the tumor microenvironment. Among the cancers of the upper GI tract, the *CHUK* gene had only demonstrated a significant negative correlation with dendritic cell infiltration in ESCA. Conversely, the expression of the *CHUK* gene was significantly negatively correlated with tumor purity in READ, indicating higher expression in the tumor microenvironment than in colorectal cancer cells. *CHUK* gene expression was also significantly positively correlated with the level of immune cell infiltration in COAD (B Cell, T CD8+, T CD4+, macrophage, neutrophil, dendritic cells) and in READ (B Cell, T CD8+, neutrophil, dendritic cells). 

In COAD and READ, the *IKBKB* gene expression level was also positively correlated with infiltration by the abovementioned immune cells. *IKBKB* expression was positively correlated with infiltrating levels of B cells in ESCA and STAD, the number of macrophages in ESCA, and of T CD4+ in STAD. 

The numbers of T CD8+, T CD4+, macrophages, and neutrophils were significantly correlated with *IKBKG* gene expression in COAD and READ. In READ, it also correlated with dendritic cells. *IKBKG* gene expression was also significantly associated with infiltrating levels of immune cells, e.g., T CD8+ in ESCA and T CD4+ and dendritic cells in STAD. These results suggest that the IKK complex may be engaged in the modulation of tumor immunity in COAD, ESCA, READ, and STAD. 

### 2.7. The Correlation between the Copy Number Variation of IKK Complex Genes and Immune Cell Infiltration in COAD, ESCA, READ, and STAD

The next step was to assess the correlation between the CNVs of IKK genes and the infiltration of immune cells in the studied cancers. This analysis was performed using TIMER-SCNA, where the CNV is categorized as deep deletion, shoulder deletion, diploid/normal, shoulder enhancement, and high enhancement. It was determined that copy number variations of the *CHUK*, *IKBKB*, and *IKBKG* genes significantly affected the infiltration of immune cells in tumors, especially in COAD and STAD (Supplementary Materials—Figures S6–S8). This may indicate that CNVs play important roles in the induction of an inflammatory response in tumor development.

### 2.8. The Influence of DNA Methylation on the mRNA Level of IKK Complex Genes in the Analyzed Cancers

**Figure 8:**
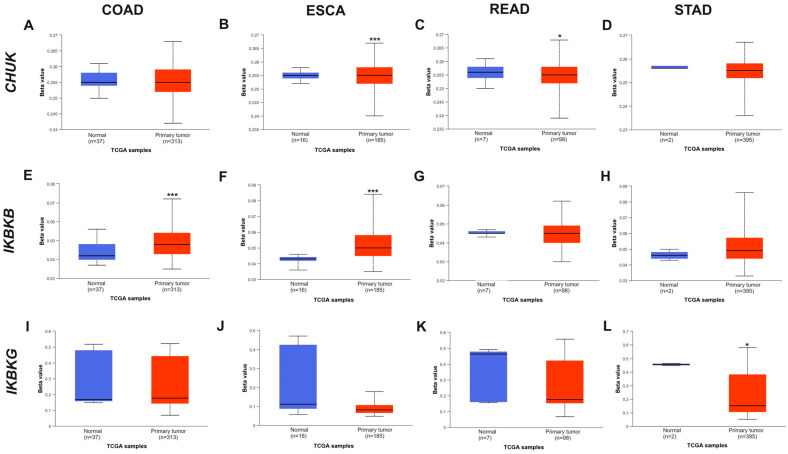
Differential promoter methylation analysis of tumor samples versus normal ones: *CHUK* gene in (**A**) COAD, (**B**) ESCA, (**C**) READ, and (**D**) STAD; *IKBKB* gene in (**E**) COAD, (**F**) ESCA, (**G**) READ, and (**H**) STAD; and *IKBKG* gene in (**I**) COAD, (**J**) ESCA, (**K**) READ, and (**L**) STAD as assessed by UALCAN analysis (the beta value indicates the level of DNA methylation ranging from 0 (unmethylated) to 1 (fully methylated); hyper-methylation (Beta value: 0.7–0.5); or hypo-methylation (Beta-value: 0.3–0.25). (* *p* < 0.05; *** *p* <0.001) Accessed 4 September 2023.

The influence of the DNA methylation status on the *IKK* complex genes in healthy tissue and COAD, ESCA, READ, and STAD tissues, as well as its association with clinicopathological factors, was assessed using the UALCAN database. In the COAD subgroup, the methylation status of the *IKBKB* gene promoter was significantly higher in cancer tissue than in healthy tissue (*p* < 0.001). In the ESCA group, the methylation status was correlated for both the *CHUK* and *IKBKB* genes (*p* < 0.001). In READ, *CHUK* promoter methylation differs between normal tissue and cancer tissue (*p* = 0.0255). In STAD, the *IKBKG* gene demonstrated significantly lower promoter methylation (*p* = 0.0350) (Figure 8). In *STAD*, all three genes were upregulated in Grade 3 (G3) compared to Grade 2 (G2) (*p* < 0.01). Other results of staging/grading/nodal metastasis involvement are presented in Supplementary Materials (Supplementary Materials—Table S1).

Generally, the MEXPRESS database identified a weak negative correlation between the mRNA expression of *CHUK*, *IKBKB*, and *IKBKG* genes and the methylation status of particular CpG islands/dinucleotides in the investigated cancers. However, a weak positive correlation for the *CHUK* gene was noted in the COAD subgroup. It is worth noting that for some regions of the *CHUK* and *IKBKG* genes, a medium level of negative correlation was found with methylation in COAD, ESCA, and READ tissues. Therefore, it appears that methylation processes may influence IKK gene expression levels. MEXPRESS analysis also indicated a medium to high positive correlation (‘r’ range 0.173–0.733) between IKK complex gene expression and CNVs, which means that genetic alterations could be activators of the transcription process. (See: Supplementary Materials—Figures S9–S14).

### 2.9. The Influence of Alterations in the IKK Complex Genes on Their Expression in the Analyzed Cancers

The cBioportal database was used to analyze alterations in IKK complex genes in the selected cancers. In the studied tumors, the frequency of gene alterations ranged from 0% to 11%. The lowest number of changes was detected in READ in all three investigated genes, and the highest was detected for the *IKBKB* gene in ESCA. The oncoprint indicates that the dominant type of alteration for all examined genes was amplification (Supplementary Materials—Figure S15). This may be related to the altered expression of these genes in COAD, ESCA, and STAD cancers (Supplementary Materials—Figures S16–S18). However, the cBioportal database did not include data on changes in the copy number or correlation of these changes with the expression of *IKBKG* gens in STAD or *CHUK*, *IKBKB*, and *IKBKG* in READ.

### 2.10. Total Protein Level of IKK Kinase Complex in COAD, ESCA, READ, and STAD

**Figure 9:**
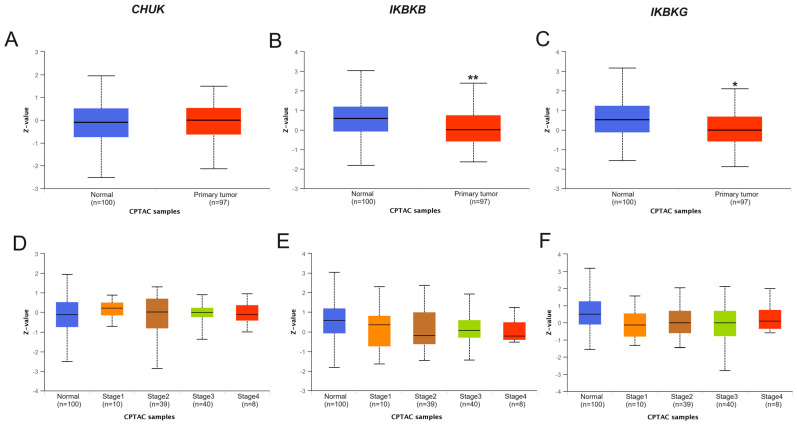
The protein level in primary tumor vs. normal tissue of (**A**) CHUK, (**B**) IKBKB, (**C**) IKBKG, and according to the individual cancer clinical stage of (**D**) CHUK, (**E**) IKBKB, and (**F**) IKBKG, in COAD based on UALCAN (* *p* < 0.05; ** *p* < 0.01; z-values—standard deviations from the median across samples for COAD; Accessed 18–19 September 2023).

The total protein levels of the IKK complex were determined using UALCAN, based on the CPTAC database. In colon cancer, lower IKBKB and IKBKG protein levels were noted in tumor tissue compared to normal tissue (*p* < 0.01; 0.036, respectively). In this type of cancer, no significant results were obtained regarding the association between the protein levels and clinical stage of disease (Figure 9). 

The UALCAN (CPTAC) database did not include any data regarding total protein levels of CHUK, IKBKB, or IKBKG in ESCA, READ, or STAD tumors.

### 2.11. Protein-Protein Interaction Analysis

Using the STRING tool, a protein-protein interaction network was constructed. The center of the network was made up of the proteins encoded by the genes of the IKK complex, i.e., CHUK, IKBKB, and IKBKG (Supplementary Materials—Figures S19–S21). The networks showed that the top 10 most functional partners for CHUK were TNFRSF1A, NFKBIA, NFKB1, IRAK1, RELA, TNF, IKBKB, TRAF6, IKBKG (scores of 0.999), and RIPK1 (score of 0.998–0.999). The top 10 for IKBKB were TNFRSF1A, NFKBIA, NFKB1, CHUK, RELA, TNF, TRAF6, IKBKG NFKBIB, and KEAP1 (all with a score of 0.997–0.999). In addition, the top 10 for IKBKG were TNFRSF1A, TAP1, NFKBIA, NFKB1, TRAF2, TANK, RIPK1, RNF31, RBCK1, and ERC1 (all with a score of 0.999). Within the generated PPI network, three clusters of interaction proteins for the studied IKK genes were identified—red, green, and blue (Supplementary Materials—Figures S22–S24). 

Interactions between all three genes in the red clusters made using the STRING tool indicate that members of this cluster are involved together in several biological processes. Most of them play an essential role in the NF-kappa-B signaling pathway, mainly through activation; here, we can distinguish the BCL10, CHUK, IKBKB, IKBKE, IKBKG, MALT1, MAVS, MYD88, TAB3, TRADD, TRAF2, TRAF5, and TRAF6 genes. Some play a central role in the regulation of cell survival, apoptosis, and induction of the latter, such as BCL10, RIPK1, TRADD, TRAF2, and TRAF5. Others are required for innate and/or adaptive immune responses, mainly BCL10, IRAK1, IRAK4, MAVS, MYD88, and RIPK2. 

In the case of the green cluster for the studied IKK complex, some proteins belong to the NF-κB signaling pathway, such as NFKB2, NFKBIB, NFKBIE, REL, and RELA. Some of the interactors are serine-threonine kinases or proteins related to them, including MAP3K1, MAP3K8, and MAP3K3. Others are related to TNF and its receptors, for example, TNF and TNFR1F1A. 

The blue cluster includes, among others, genes related to the NF-κB, mTOR, and AKT pathways, as well as molecular chaperones such as Hsp90 and CDC37.

## 3. Discussion

The malignant tumors of the GI tract are one of the most common cancers in the world. While all can be influenced by lifestyle and the living environment, many have also been found to be significantly influenced by changes in genes and proteins at the molecular level [2]. The IKK kinase complex consists of two enzymatic subunits encoded by the *CHUK* and *IKBKB* genes and a regulatory subunit encoded by the *IKBKG* (*NEMO*) gene; the complex as a whole is strictly connected with the NF-kB signaling pathway, where it helps the NF-kB transcription factor to enter the cell nucleus and activate various genes. As such, changes in these genes may affect cancer development; however, the role of the *CHUK*, *IKBKB*, and *IKBKG* genes is not fully known [28]. According to our knowledge, the present study is the first comprehensive assessment of the potential role of these genes in the development and progression of selected GI cancers, their interactions with tumor-infiltrating immune cells, and their influence on patient survival.

Our findings indicate that the *CHUK*, *IKBKB*, and *IKBKG* genes demonstrated varied expression in the tested GI cancers. *IKBKB* and *IKBKG* were both upregulated in all examined cancers, while *CHUK* only presented increased expression in ESCA and STAD. Hence, it appears that changes in *IKK* gene expression may be related to the development of analyzed cancers.

It is difficult to compare our studies to others on selected gastrointestinal cancers, as although tested genes have been examined in various diseases, they have often been studies performed on cell lines or animal models. Additionally, most previous studies assessed only one or two genes from the IKK complex at once. For example, Hu et al. reported a higher expression of the *IKBKB* gene and its protein in patients with epithelial ovarian cancer compared to the control group [29]. In addition, Sun et al. found IKBKB and IKBKG protein levels to be higher in kidney renal clear cell carcinoma tumor tissue than in normal tissue [30]. This may indicate the IKK complex, and especially the *IKBKB* gene, in the development of cancer. However, Mieczkowski et al. reported lowered *IKBKB* gene expression in glioblastoma, especially compared to normal tissue and low-grade tumors, such as juvenile pilocytic astrocytoma. This may be related to the immunoreactivity of the tested cancer tissue and tumor-infiltrating macrophages, which may impair anti-tumor immune responses and favor glioblastoma progression [31]. The important roles of the tumor microenvironment and antitumor immune response seem to be confirmed by the fact that, in a mouse model, constitutively active IKBKB in the T-lymphocytes allowed the restoration of NF-κB pathway activity in these T-cells, thanks to which they exerted anti-tumor activity and controlled tumor growth. The IKBKB may contribute to antitumor immunity through the activation of T-lymphocytes or by increased production of anti-inflammatory cytokines by dendritic cells [32,33].

Our results also showed that the *IKK* gene complex may be engaged in the modulation of tumor immunity in COAD, ESCA, READ, and STAD. The potential mechanism may be associated with the increased degradation of inhibitory NF-κB dimers that would lead to the activation of dependent pathways and, *inter alia*, excessive cell proliferation. For example, data indicate that a reduction in CHUK protein level contributes to the simultaneous induction of apoptosis [34,35]. Additionally, this theory is supported by the fact that the upregulation of CHUK leads to the increased proliferation and inhibition of apoptosis in mantle cell lymphoma [36]. A similar connection was also demonstrated in non-small cell lung cancer cells, which demonstrated elevated *CHUK* mRNA and protein expression compared to normal cells. Additionally, it was shown that increased expression was associated with excessive cell proliferation, and its knockdown had the opposite effect [37]. 

Other research on IKBKB found that it can promote the growth of breast cancer cells, although this was only investigated at the protein level [38]. Therefore, these studies confirm that *IKK* complex genes may influence the development of cancer, probably through stimulation of the NF-κB pathway and/or other signaling pathways. In the present study, the STRING tool indicated that the protein products of the analyzed genes interacted with proteins of various signaling pathways associated with *inter alia* toll-like receptors, TNF-mediating signaling, and MAPK pathways. Sakamoto et al. found that *IKBKB* regulates gastric carcinogenesis in animal models through the expression of IL-1α, which is associated with anti-apoptotic signaling and cell proliferation [39]. On the other hand, Page et al. found that *IKBKB* acted as a skin cancer suppressor via p16 and p19, again in an animal model. The absence of p16 and p19, leading to an increase in IKBKB expression, favors the appearance of a highly aggressive kind of skin cancer [40]. It can be concluded that the IKK complex is an important factor during carcinogenesis, and its role depends on the molecular context in a given cell and the signaling pathway it activates. 

The association of the studied genes with proteins included in the clusters, obtained by STRING, may show their role in proliferation, differentiation, cell survival, or potential anti-inflammatory response through their interaction with other signaling pathways. 

Some of the genes from the cluster network for IKK are placed on the same chromosome, which may indicate that they are regulated together and are thus also dysregulated together in cases of tumor development. For example, from the red cluster, IKBKG, IRAK1, and TAB3 are placed together on Chromosome X, or IKBKB and RIPK2 on Chromosome 8. In the green cluster, there are the genes MAP3K8, PRKCQ, and PTEN, which are located at the same chromosome 10 as the CHUK gene. However, the theory assuming their co-expression or joint regulation requires experimental verification, as previous reports have rather indicated their functional relationship in signaling pathways. For example, Zaidi et al. showed that PTEN, in response to the action of the causative agent profilin, had a physical effect and inhibited the phosphorylation of IKK, consequently leading to a decrease in the activity of NF-κB-dependent genes involved in cancer cell proliferation [41]. In the study by Qiang et al., it was suggested that IFNα silenced the expression of carcinogen COX2 in bladder cancer by inhibiting the NF-κB pathway via MAP3K8 [42]. 

Additionally, the blue cluster also contains functionally related genes. The results obtained so far suggest that cell metabolism, proliferation, survival, growth, and angiogenesis may be regulated by products of CHUK, IKBKB, and IKBKG genes, which, together with the others, form a core gene network of interactions. AKT1, which is one of three closely related serine/threonine-protein kinases regulating cell growth, appears to be at the center of this complex network. The Cdc 37 protein (encoded by the CDC37 gene), as well as Hsp90, is a molecular chaperone that forms a complex with the kinase domain of IKKalpha/IKKbeta. Hsp90 has four isoforms, of which the isoform encoded by the HSP90AA1 gene is the most important in IKK signal transduction. The second of them (the product of the HSP90AB1 gene) could bind to LRP5 and inhibit its ubiquitin-mediated degradation and subsequently activate not only the AKT but also the Wnt/β-catenin signaling pathways [43]. Thus, CHUK, IKBKB gene products, and co-chaperones FKBP5 (FKBP51), TSC1, and TSC2 interact with Hsp90 and behave like tumor suppressors, while AKT1 shows oncogenic behavior and is involved in the enhanced activity of many signaling pathways. mTOR remains a key target for Akt to promote tumorigenesis [44]. The current status of the PTEN function may be important in determining the direction of observed regulatory changes [45]. The activity of FOXOs, transcription factors that are involved in the regulation of several cell-death-related genes, depends on interaction with IKK. This kinase immobilizes FOXOs (e.g., FOXO1 or FOXO3) in cytosol and inactivates their transcriptional activity by phosphorylation [46,47]. In the blue cluster of the IKBKG, the genes encoding various regulatory subunits responsible for the recruitment of subsequent regulatory factors are assigned. The genes included in this cluster are largely associated with the T-cell receptor signaling pathway. Finally, the IKK kinase complex is the core element in the regulation of NF-κB-dependent gene transcription.

The changes in the expression of the analyzed genes could result from various genetic and epigenetic processes such as CNV, a chromosomal instability of the methylation status. Regarding genetic alterations, it was observed that the dominant change for all three genes in all types of investigated cancers was amplification; therefore, it can be assumed that a higher gene copy number was associated with an increased risk of cancer. Thu et al. also reported that *IKBKB* expression was increased in cells, demonstrating a gain in copy number compared to those that did not [48]. Furthermore, other studies, have found that mutations leading to the deletion of gene fragments or loss of *IKBKG* function were associated with an inhibition of the NF-κB pathway and activation of apoptosis, which could have a protective function against the development of cancer [49,50,51,52]. However, in the case of CNVs, it was found that some of the investigated cancer types had a significant influence on the induction of the inflammation response of the immune system during tumor development, which is a positive aspect of these genetic changes. 

*CHUK*, *IKBKB*, and *IKBKG* differed with regard to the gene promoter methylation status. The key findings indicated that, generally, the *IKBKB* promoter was hypermethylated in COAD and ESCA. The *IKBKG* gene promoter was strongly hypomethylated in STAD. The slight but significant hypomethylation of the *CHUK* gene was present in ESCA, READ, and STAD. In most of the analyzed cancers, this was associated with the increased expression of these genes in cancer tissues. Maeda et al. reported the opposite results, where hypermethylation of the *CHUK* gene promoter was demonstrated and, consequently, reduced the expression of its mRNA as well as lower immunoreactivity of the encoded protein, which in this case was associated with the progression of oral cancer [53]. 

Simultaneously, in our results, the increase in *IKBKB* expression strongly correlates with the increased CNVs. Therefore, the methylation status seems to not be the main and only factor contributing to changes in *IKK* complex gene expression. It is likely that CNVs have a greater influence on *CHUK*, *IKBKB*, and *IKBKG* gene upregulation and thus the activation of transcriptional processes. Therefore, our findings indicate that both CNVs and methylation level are crucial factors in regulating *IKK* complex gene expression. These processes result in changes in gene expression, and abnormalities remain in direct correlation with clinicopathological parameters and demographic features in all investigated types of GI cancers, which can be potentially used in clinical practice. 

No significant trends were noted between clinical staging and the expression of any genes in the investigated cancers; however, *IKBKG* gene expression was found to be decreased in the advanced stages of STAD. In addition, the studied genes, especially *CHUK* and *IKBKB*, were upregulated in cancers with a higher degree of histological malignancy and a greater degree of lymph node infiltration, suggesting they may also be associated with cancer progression. This may be confirmed by the fact that, in the case of ovarian cancer, *IKBKB* expression was upregulated in cases of poor differentiation in histological grade and with the presence of lymph node metastasis [29]. Bennett et al. reported that high *CHUK* expression was connected with the histological grade of breast cancer [35].

Our findings also indicate that the genes of the IKK complex may play a role in the overall survival of patients with the analyzed GI cancers; more specifically, their reduced expression was associated with a shorter OS. This relationship has been confirmed in gastric cancer by Gayed et al., where favorable OS was found to be significantly correlated with *CHUK* expression, but with worse OS with *IKBKB* and *IKBKG* gene expression [54]. In contrast, in breast cancer, Bennett et al. showed that increased *CHUK* expression was not associated with overall survival but had a significant effect on recurrence-free survival after treatment [35]. This may indicate that *CHUK* overexpression is an unfavorable predictor of breast cancer treatment with tamoxifen, associated with a shortened time to cancer recurrence.

The results obtained in this study will be verified in the near future during original research in groups of patients with COAD, ESCA, READ, and STAD. Additionally, performing functional tests through in vitro studies on cell lines derived from these selected gastrointestinal cancers will allow for an assessment of the influence of *IKK* complex genes and other factors, including potential drugs, on the expression of these genes as well as on the process of cancer development and progression. All these things will allow for a determination of the final conclusions and confirm whether the analyzed *IKK* genes can be used in clinical practice as prognostic/predictive factors or the targets of individualized therapy.

## 4. Materials and Methods

### 4.1. The Expression of the IKK Genes Complex in Cancer vs. Normal Tissues

The expression of the *IKK* complex members in cancerous and corresponding normal tissues was compared using the Tumor Immune Estimation Resource 2.0 (TIMER 2.0) (http://timer.cistrome.org/, accessed on 16 January 2023) database. The expression of three selected genes in cancer tissue compared to normal tissue was assessed using the Gene_DE module. The data given as boxplots compare the differential gene expression of various tumor types with their adjacent non-cancerous tissues (when information is available); the data are taken from The Cancer Genome Atlas (TCGA) project. Statistical significance was assessed using the Wilcoxon test, and *p*-values are marked with asterisks: *: *p* < 0.05; **: *p* < 0.01; ***: *p* < 0.001 [55]. 

Data contained in the UALCAN database (University of Alabama at the Birmingham Cancer data analysis portal, https://ualcan.path.uab.edu/, accessed on 17 January 2023) [56,57] were used to generate individual box-whisker plots illustrating the *CHUK*, *IKBKB*, and *IKBKG* expression in normal and cancer tissues in patients with COAD, ESCA, READ, and STAD. Statistical significance was assessed using Welch’s *t*-test, with a *p*-value < 0.05 indicating a significant value. 

The Gene Expression Profiling Interactive Analysis 2 (GEPIA2) database (http://gepia2.cancer-pku.cn/#index, accessed on 17 January 2023) [58] was also used to assess *CHUK*, *IKBKB*, and *IKBKG* gene expression level differences between normal and tumor tissues in COAD, ESCA, READ, and STAD patients. The data are taken from TCGA and GTEx (genotype-tissue expression portal) projects. The *p*-values were calculated by one-way ANOVA, the disease state (tumor or normal) was taken as the variable for calculating differential expression, and box plots were received using the Expression DIY tool. A *p*-value of <0.01 was considered statistically significant. 

### 4.2. The mRNA Expression in the IKK Genes Complex in COAD, ESCA, READ, and STAD Cancers According to Clinicopathological Features

Data contained in the UALCAN database (https://ualcan.path.uab.edu/, accessed on 17–19 January 2023) [56,57] were used to generate individual box-whisker plots illustrating *CHUK*, *IKBKB*, and *IKBKG* expression in patients with COAD, ESCA, READ, and STAD with regard to selected clinicopathological factors. The following characteristics were included in the analysis: histological type, stage, grade, nodal metastases, weight, alcohol consumption, smoking, and *H. pylori* infection status. Statistical significance was assessed using Welch’s *t*-test (*p* < 0.05).

### 4.3. The Methylation Status, Copy Number Variations, and Expression of IKK Gene Complexes in COAD, ESCA, READ, and STAD Cancers

The UALCAN database (https://ualcan.path.uab.edu/, accessed on 14 September 2023) [56,57] was used to evaluate the influence of DNA methylation status on the expression of *IKK* complex genes in healthy tissue and COAD, ESCA, READ, and STAD tumors. The analysis also included associations with clinicopathological factors (stage, grade, and nodal metastases). The DNA methylation level was presented as the beta value, ranging from 0 (unmethylated) to 1 (fully methylated). Cut-off values to indicate hyper-methylation [0.7–0.5] or hypo-methylation [0.3–0.25] were established [59,60].

Additionally, TCGA gene expression, DNA methylation, and copy number data, as well as relation to genomic location, were assessed using the MEXPRESS online tool (https://mexpress.ugent.be/index.html, accessed between 16–18 October 2023), which enabled the assessment of [61,62]. MEXPRESS generates individual plots and calculates their statistical significance. The *p*-values were determined by *t* test or ANOVA (Benjamini-Hochberg-adjusted) and Pearson correlation coefficients (r) were shown for two numerical variables.

### 4.4. Gene Alterations and Their Influence on IKK Complex Gene Expression Level

cBioportal (https://www.cbioportal.org/, accessed between 11–13 September 2023) is an online database used to evaluate putative copy number alterations, linear copy number values, and other genetic aberrations for selected genes [63,64,65]. The TCGA Firehose Legacy database was used for COAD, ESCA, and STAD, and MSK Nature Medicine 2022 was used for READ. An oncoprint plot was generated for individual *IKK* complex gene members.

### 4.5. Survival Analysis

The Kaplan–Meier plotter (https://kmplot.com/analysis/, accessed between 4–5 September 2023) was used to assess the prognostic value of *CHUK*, *IKBKB*, and *IKBKG* gene expression (low vs. high expression) for overall survival (OS) in COAD, ESCA, READ, and STAD cancers [66]. The KM plotters were drawn using an auto-selected threshold and best cutoff, with an array quality control to “exclude biased arrays”. Patients were divided according to the median value of *IKK* gene expression. The distinct *p*-values between the two subgroups were calculated using the log-rank test. A *p*-value < 0.05 was considered statistically significant.

### 4.6. Correlation between the Degree of Tumor Infiltration by Immune Cells and IKK Gene Expression in COAD, ESCA, READ, and STAD Cancers

Interactions between studied gene expression and immune cell infiltration (CD4+T-cells, CD8+T-cells, B-cells, neutrophils, macrophages, and dendritic cells) in the tumor microenvironment of COAD, ESCA, READ, and STAD were evaluated by using the module “Immune Association-Gene” in the TIMER 2.0 database (http://timer.cistrome.org/, accessed between 6–8 February 2023) [55]. The scatterplots show the correlation between gene expression and immune cell infiltration degree with the purity-corrected partial Spearman’s rho value and statistical significance.

### 4.7. Correlation between CNV and Immune Infiltration

This analysis was performed using TIMER-SCNA (Somatic Copy Number Alterations) (http://timer.cistrome.org/, accessed on 6–8 February 2023) [55], where the CNV is categorized as: deep deletion, shoulder deletion, diploid/normal, shoulder enhancement, and high enhancement. The generated boxplots indicate the distributions of each immune subset at each copy number status in a chosen cancer type. The infiltration level for each SCNA category was compared with the values for normal cells using a two-tailed Wilcoxon rank sum test. The *p*-values are marked with asterisks: *: *p* < 0.05; **: *p* < 0.01; ***: *p* < 0.001.

### 4.8. The Total Protein Level of the IKK Members Complex in COAD, ESCA, READ, and STAD Cancers

The protein expression of CHUK, IKBKB, and IKBKG in COAD and normal tissue was determined using mass-spectrometry-based proteomic data retrieved from the Clinical Proteomic Tumor Analysis Consortium (CPTAC) and the International Cancer Proteogenome Consortium (ICPC) datasets through the UALCAN website (http://ualcan.path.uab.edu/index.html, accessed on 18–19 September 2023) [56,67,68]. The protein data in ESCA, READ, or STAD tissues were not available.

### 4.9. Protein-Protein Interactions (PPI) between Individual IKK Complex Members

The STRING database (https://string-db.org/, accessed between 6–7 November 2023 and 15–18 March 2024) is an online tool for the evaluation of known and predicted protein-protein interactions [69]. It enables the assessment of direct—physical and indirect—functional associations. In the current analysis, 50 interactions with a minimum confidence at level 0.9 were predicted for *IKK* complex members. The k-means clustering of the generated PPI networks for IKK was performed with a pre-set of three clusters (marked as red, green, and blue).

Unless otherwise specified, in all analyses, a *p*-value < 0.05 was considered significant. All the box-whisker plots present median, minimum, and maximum values, 1st and 3rd quartiles.

## 5. Conclusions

Our bioinformatics analysis indicates that *CHUK*, *IKBKB*, and *IKBKG* upregulation may be connected with the development and progression of selected gastrointestinal cancers. In particular, changes in *CHUK* and *IKBKB* may be associated with the faster progression of these cancers. In the case of *CHUK*, its expression was decreased in more advanced cancer and in the presence of lymph node metastases in ESCA cancer. For *IKBKB*, the results were the opposite; for example, in READ, its expression was higher in more advanced cancer, and in STAD, its expression was higher in the presence of lymph node metastases. In ESCA, all three genes studied were associated with patients’ weight and alcohol consumption, and *IKBKG* was associated with tobacco smoking. In STAD, the reduced expression of the *CHUK* gene was associated with *H. pylori* infection. 

Interestingly, the increased expression of IKK genes is probably the result of a gain in the CNVs of these genes. For all IKK complex genes, there was amplification, which may translate into a change in their expression in GI cancers. Furthermore, the promoter methylation status of the studied genes may be associated with changes in the expression of these genes. Including the increased promoter methylation of the genes: CHUK in ESCA, READ and IKBKB in COAD, and ESCA and IKBKG in STAD. On the other hand, they may influence the process of carcinogenesis through various signaling pathways where the investigated complex may be potentially involved. The STRING analysis showed that IKK genes are related to several biological processes: cell survival, apoptosis, immune response, and the signaling pathways mTOR and AKT.

An interesting target for further research seems to be *IKBKG* as a regulator of the activity of the entire IKK complex and *CHUK* as a subunit of the kinase involved in both the canonical and non-canonical NF-κB pathway. Even more so, since our bioinformatics analyses showed that the reduction of *CHUK* and *IKBKG* expression may be a negative prognostic factor in STAD, the downregulation of *IKBKG* may be a positive prognostic factor in ESCA.

In summary, IKK genes, especially *CHUK* and *IKBKG*, have an important role in the development and progression of selected gastrointestinal cancers; they may serve as potential prognostic markers and therapeutic targets for analyzed tumors useful in clinical practice.

## Figures and Tables

**Table 1:** The comparison of *CHUK*, *IKBKB,* and *IKBKG* gene expression according to histological type or subtype of: COAD, ESCA, READ, and STAD cancers. The value of *p* < 0.05 was statistically significant (based on UALCAN accessed 17–19 January 2023).

		*CHUK*	*IKBKB*	*IKBKG*
Type of Cancer	Histologic Type/Subtype Comparison	*p*	*p*	*p*
COAD	Adenocarcinoma-vs.-Mucinous-adenocarcinoma	0.7001	0.4100	0.3987
ESCA	Adenocarcinoma-vs.-Squamous-cell-carcinoma	0.0000	0.6031	0.0000
READ	Adenocarcinoma-vs.-Mucinous-adenocarcinoma	0.5336	0.1156	0.4641
STAD	Adenocarcinoma (NOS)-vs.-Adenocarcinoma (Diffuse)	0.2487	0.2596	0.5854
	Adenocarcinoma (NOS)-vs.-Adenocarcinoma (Signet Ring)	0.7736	0.6325	0.7628
	Adenocarcinoma (NOS)-vs.-IntestinalAdenocarcinoma (NOS)	0.9384	0.5497	0.7661
	Adenocarcinoma (NOS)-vs.-IntestinalAdenocarcinoma (Tubular)	0.0686	0.4179	0.1598
	Adenocarcinoma (NOS)-vs.-IntestinalAdenocarcinoma (Mucinous)	0.9272	0.8843	0.5188
	Adenocarcinoma (NOS)-vs.-IntestinalAdenocarcinoma (Papillary)	0.4362	0.6940	0.3198
	Adenocarcinoma (Diffuse)-vs.-Adenocarcinoma (Signet Ring)	0.7821	0.9570	0.9383
	Adenocarcinoma (Diffuse)-vs.-IntestinalAdenocarcinoma (NOS)	0.2785	0.1394	0.3986
	Adenocarcinoma (Diffuse)-vs.-IntestinalAdenocarcinoma (Tubular)	0.0103	0.9399	0.3798
	Adenocarcinoma (Diffuse)-vs.-IntestinalAdenocarcinoma (Mucinous)	0.5601	0.4029	0.2754
	Adenocarcinoma (Diffuse)-vs.-IntestinalAdenocarcinoma (Papillary)	0.7246	0.8416	0.3189
	Adenocarcinoma (Signet Ring)-vs.-IntestinalAdenocarcinoma (NOS)	0.7430	0.4773	0.6611
	Adenocarcinoma (Signet Ring)-vs.-IntestinalAdenocarcinoma (Tubular)	0.2503	0.9971	0.4174
	Adenocarcinoma (Signet Ring)-vs.-IntestinalAdenocarcinoma (Mucinous)	0.8460	0.5944	0.4367
	Adenocarcinoma (Signet Ring)-vs.-IntestinalAdenocarcinoma (Papillary)	0.5904	0.8040	0.4677
	IntestinalAdenocarcinoma (NOS)-vs.-IntestinalAdenocarcinoma (Tubular)	0.1284	0.2499	0.0943
	IntestinalAdenocarcinoma (NOS)-vs.-IntestinalAdenocarcinoma (Mucinous)	0.8944	0.8341	0.7244
	IntestinalAdenocarcinoma (NOS)-vs.-IntestinalAdenocarcinoma (Papillary)	0.5904	0.8040	0.4677
	IntestinalAdenocarcinoma (Tubular)-vs.-IntestinalAdenocarcinoma (Mucinous)	0.2637	0.4494	0.0710
	IntestinalAdenocarcinoma (Tubular)-vs.-IntestinalAdenocarcinoma (Papillary)	0.1551	0.7548	0.6432
	IntestinalAdenocarcinoma (Mucinous)-vs.-IntestinalAdenocarcinoma (Papillary)	0.5283	0.6703	0.3152

## Data Availability

The datasets used in this study are available publicly, links to the archives: http://timer.cistrome.org/ (accessed on 16 January 2023; 6–8 February 2023), http://gepia.cancer-pku.cn/ (accessed on 17 January 2023) https://ualcan.path.uab.edu/analysis.html (accessed on 17–19 January 2023; 14 September 2023; 18–19 September 2023), https://kmplot.com/analysis/ (accessed on 4–5 September 2023), http://www.cbioportal.org/ (accessed on 11–13 September 2023), https://mexpress.ugent.be/ (accessed on 16–18 October 2023), https://string-db.org/ (accessed on 6–7 November 2023; 15–18 March 2024). All details are described in Section 4.

## Supplementary Materials

The following supporting information can be downloaded at: https://www.mdpi.com/article/10.3390/ijms25189868/s1.
