# Supplementary material for: Bioinformatic Analysis of IKK Complex Genes Expression in Selected Gastrointestinal Cancers
Source: Int J Mol Sci. 2024 Sep 12;25(18):9868. doi: 10.3390/ijms25189868 (PMC11432643; doi:10.3390/ijms25189868)

Supplementary materials - Figure S1. Differences of *IKK* complex genes expression according to patient`s weight: A) *CHUK* gene, B) *IKBKB* gene, C) *IKBKG* gene in COAD; D) *CHUK* gene, E) *IKBKB* gene F) *IKBKG* gene in ESCA; G) *CHUK* gene, H) *IKBKB* gene I) *IKBKG* gene in READ based on UALCAN web tool (normal weight - BMI greater than equal to 18.5 and BMI less than 25; extreme weight - BMI greater than equal to 25 and BMI less than 30; obese - BMI greater than equal to 30 and BMI less than 40; extreme obesity - BMI greater than 40). Access 17-19.02.2023.

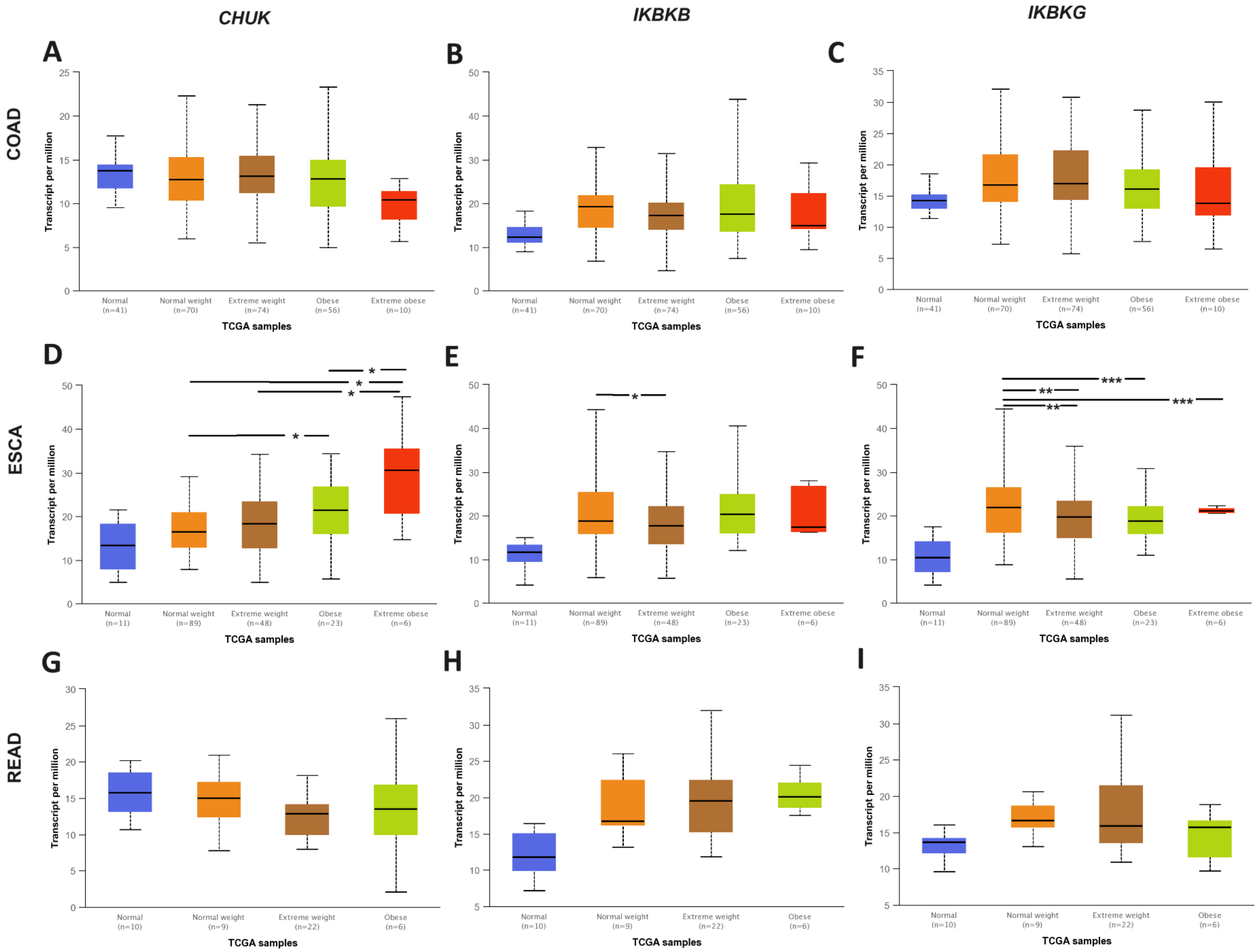

Supplement: Supplementary file 1 [file ijms-25-09868-s001.zip › Supplementary materials - Figure S1.pdf]
