# Supplementary material for: Bioinformatic Analysis of IKK Complex Genes Expression in Selected Gastrointestinal Cancers
Source: Int J Mol Sci. 2024 Sep 12;25(18):9868. doi: 10.3390/ijms25189868 (PMC11432643; doi:10.3390/ijms25189868)

Supplementary materials - Figure S10. The correlation between *CHUK* gene expression and DNA methylation status in READ and STAD, based on MEXPRESS web tool (access 16-18.10.2023). The left part of every chart shows gene together with its transcripts, CpG islands and all the individual CpG dinucleotides. The samples are ordered by their expression value. The horizontal lines shows the correlation between DNA methylation and gene expression. Statistical significance was indicated in the right side of every chart by using asterisks.

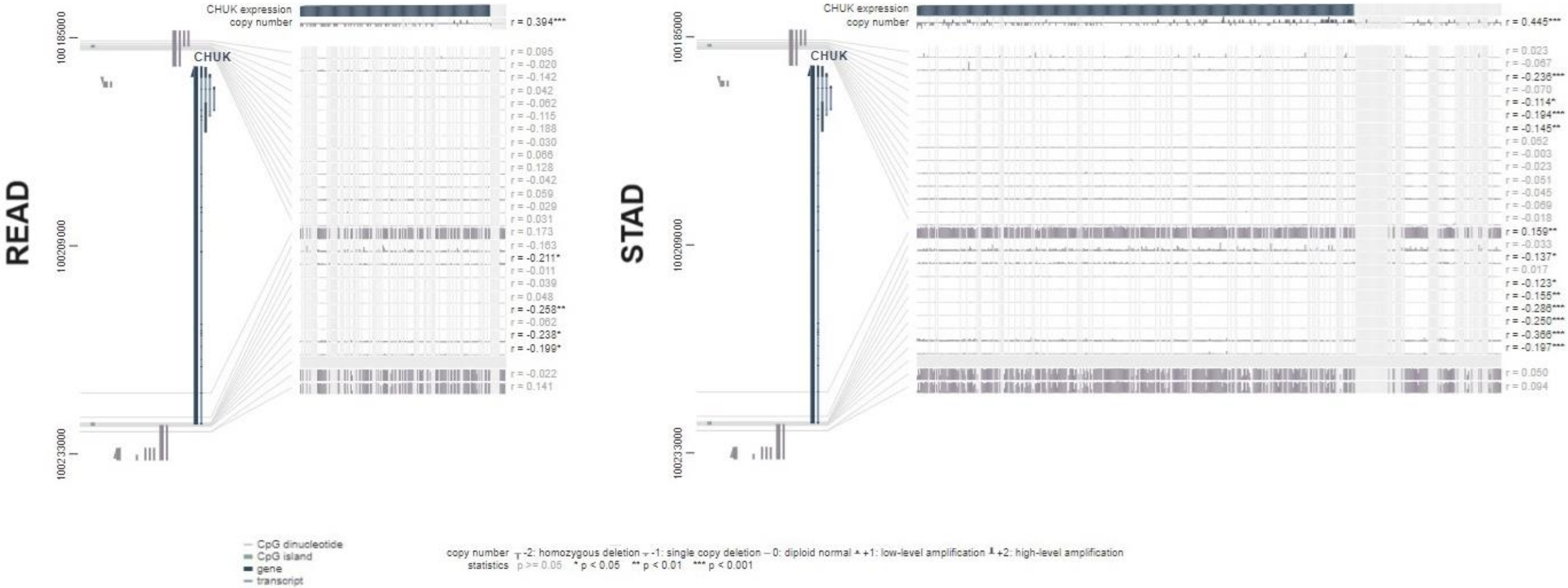

Supplement: Supplementary file 1 [file ijms-25-09868-s001.zip › Supplementary materials - Figure S10.pdf]
