# Supplementary material for: Bioinformatic Analysis of IKK Complex Genes Expression in Selected Gastrointestinal Cancers
Source: Int J Mol Sci. 2024 Sep 12;25(18):9868. doi: 10.3390/ijms25189868 (PMC11432643; doi:10.3390/ijms25189868)

Supplementary materials - Figure S15. The overall genetic alteration of *IKK* complex genes in A) COAD, B) ESCA, C) READ, D) STAD based on cBioportal (access 11-13.09.2023).

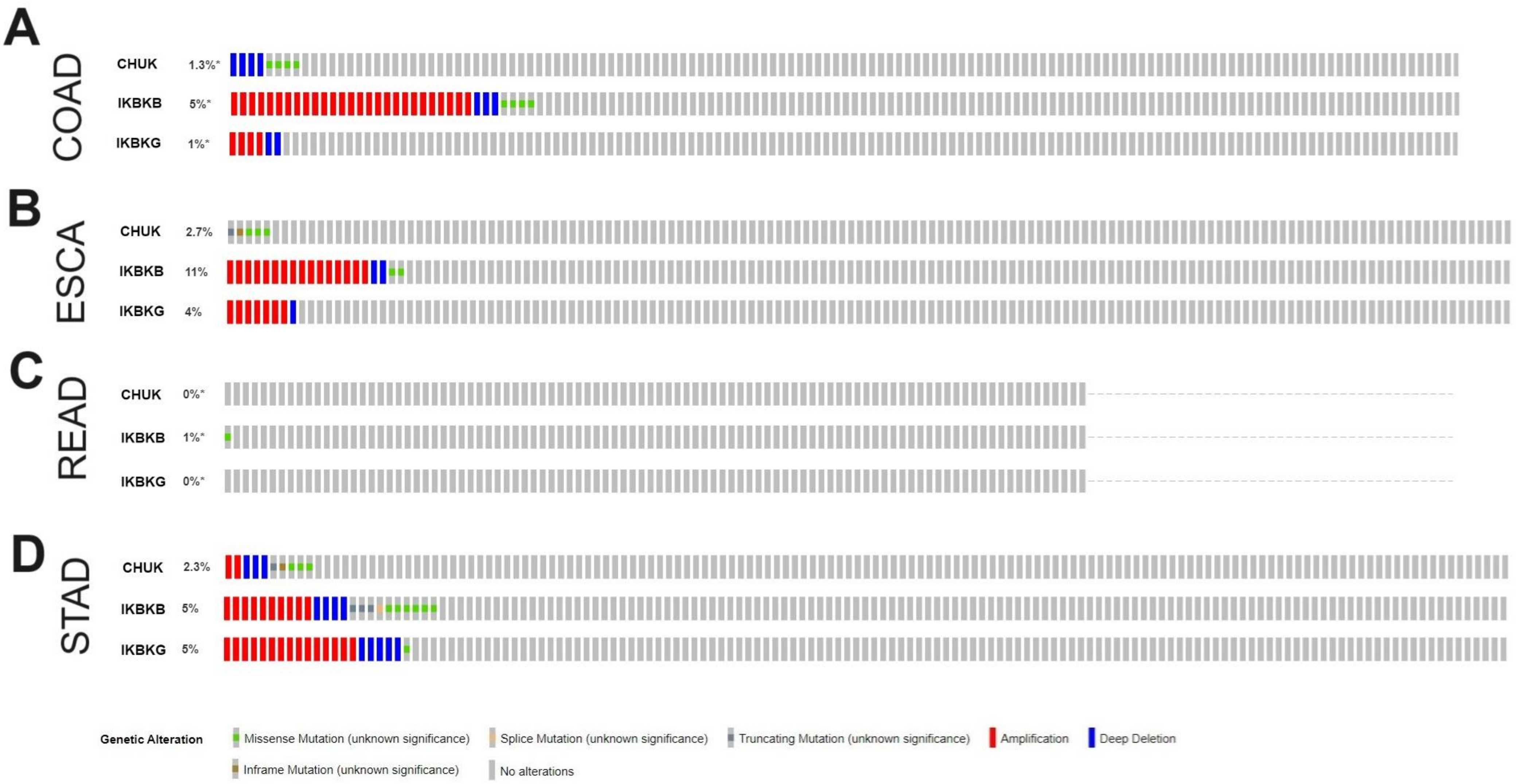

Supplement: Supplementary file 1 [file ijms-25-09868-s001.zip › Supplementary materials - Figure S15.pdf]
