# Supplementary material for: Bioinformatic Analysis of IKK Complex Genes Expression in Selected Gastrointestinal Cancers
Source: Int J Mol Sci. 2024 Sep 12;25(18):9868. doi: 10.3390/ijms25189868 (PMC11432643; doi:10.3390/ijms25189868)

Supplementary materials - Figure S18. The association between the expression of IKK complex genes and putative copy number alteration as well as the correlation between genes expression and linear copy number values in STAD based on cBioportal (access: 11-13.09.2023). The cBioportal database did not contain any data on the IKBKG gene in STAD and *CHUK, IKBKB* and *IKBKG* in READ.

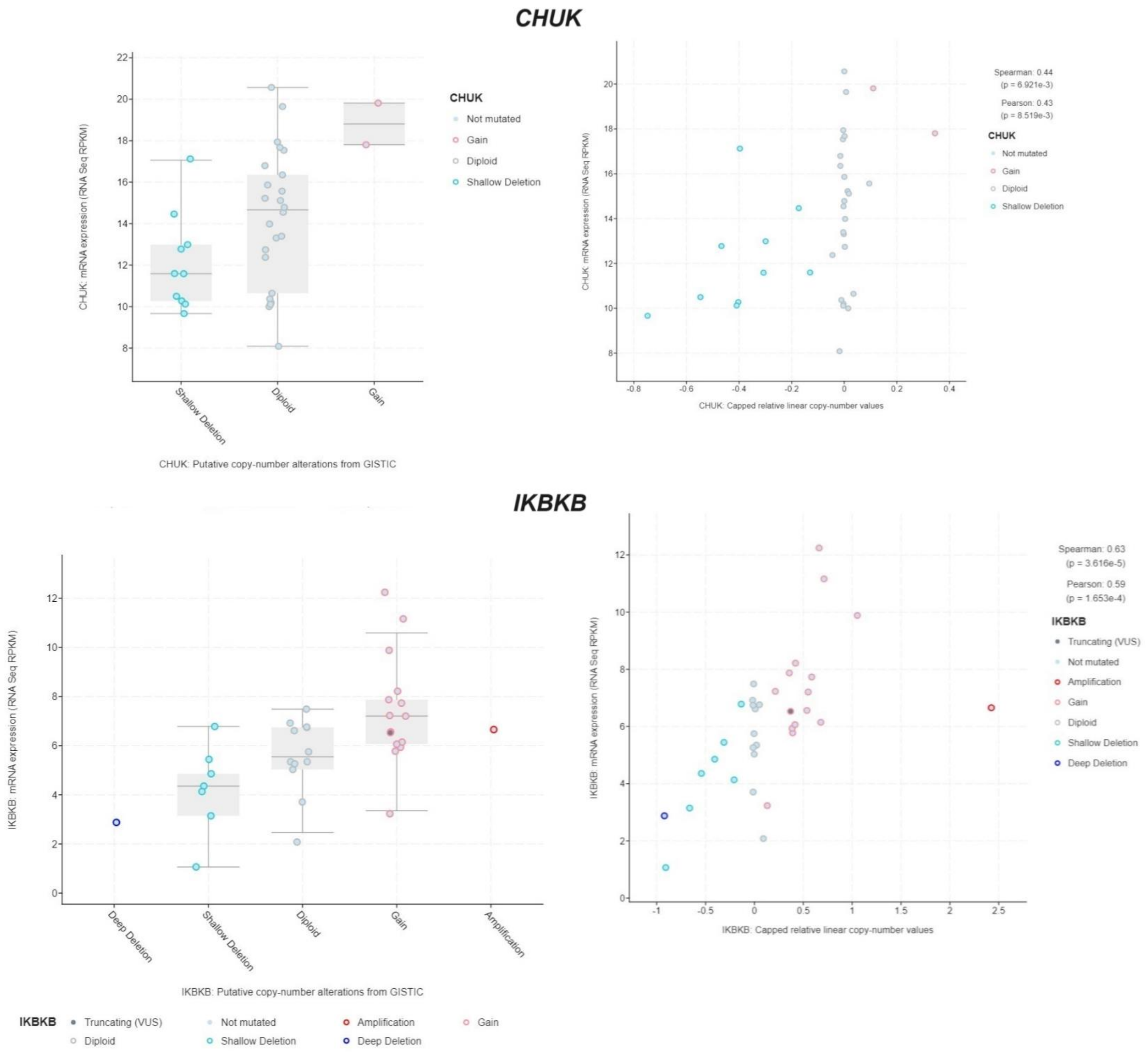

Supplement: Supplementary file 1 [file ijms-25-09868-s001.zip › Supplementary materials - Figure S18.pdf]
