# Supplementary material for: Bioinformatic Analysis of IKK Complex Genes Expression in Selected Gastrointestinal Cancers
Source: Int J Mol Sci. 2024 Sep 12;25(18):9868. doi: 10.3390/ijms25189868 (PMC11432643; doi:10.3390/ijms25189868)

Supplementary materials - Figure S2. Differences of *IKK* complex genes expression according to : A) frequency of alcohol consumption by patients with ESCA; B) smoking of tobacco by patients with ESCA; C) *Helicobacter pylori* infection status in patients with STAD based on UALCAN web tool. Access 17-18.01.2023.

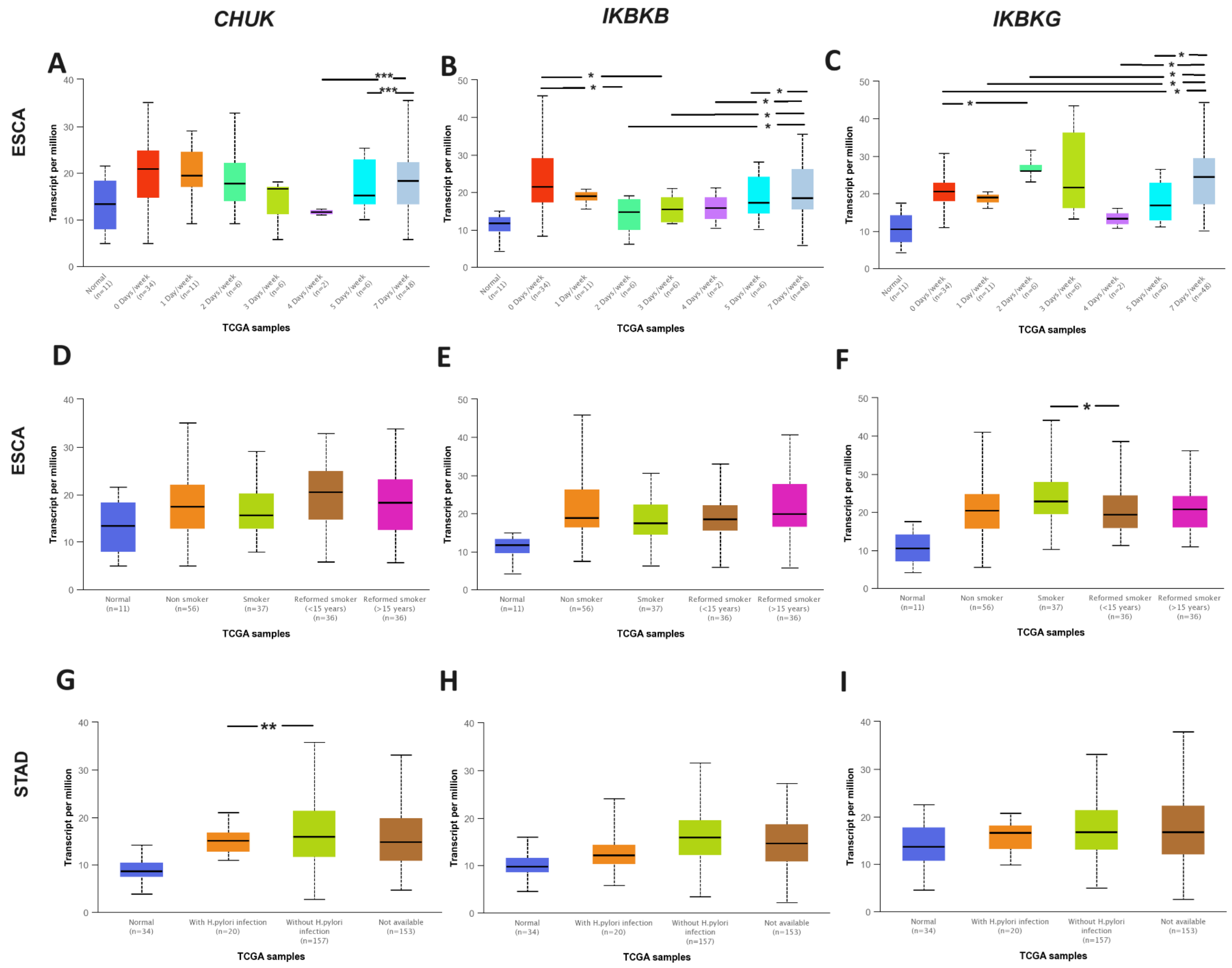

Supplement: Supplementary file 1 [file ijms-25-09868-s001.zip › Supplementary materials - Figure S2.pdf]
