# Supplementary material for: Bioinformatic Analysis of IKK Complex Genes Expression in Selected Gastrointestinal Cancers
Source: Int J Mol Sci. 2024 Sep 12;25(18):9868. doi: 10.3390/ijms25189868 (PMC11432643; doi:10.3390/ijms25189868)

Supplementary materials - Figure S21. The protein-protein interaction network of IKBKG based on STRING database (access: 06-07.11.2023). For all three proteins, 50 interactors, with a minimum confidence at level of 0.9, were predicted (for all PP enrichment p- value 1.06×10^{-16}).

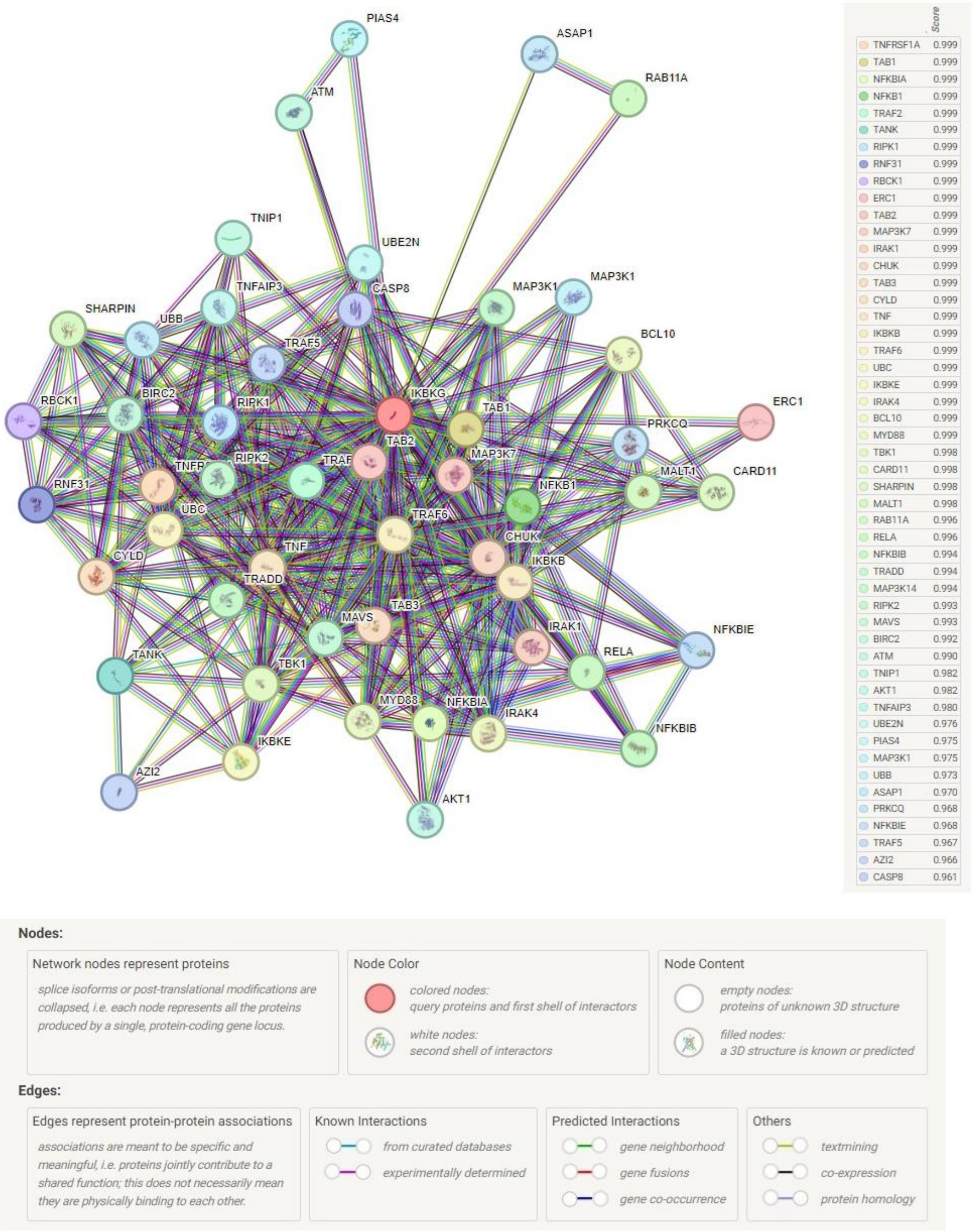

Supplement: Supplementary file 1 [file ijms-25-09868-s001.zip › Supplementary materials - Figure S21.pdf]
