# Supplementary material for: Bioinformatic Analysis of IKK Complex Genes Expression in Selected Gastrointestinal Cancers
Source: Int J Mol Sci. 2024 Sep 12;25(18):9868. doi: 10.3390/ijms25189868 (PMC11432643; doi:10.3390/ijms25189868)
Supplement: Supplementary file 1 [file ijms-25-09868-s001.zip › Supplementary materials - Figure S22.pdf]

Supplementary materials - Figure S22. The cluster analysis for CHUK by using STRING database (access 15-18.03.2024). The identified clusters are marked as red, green and blue.

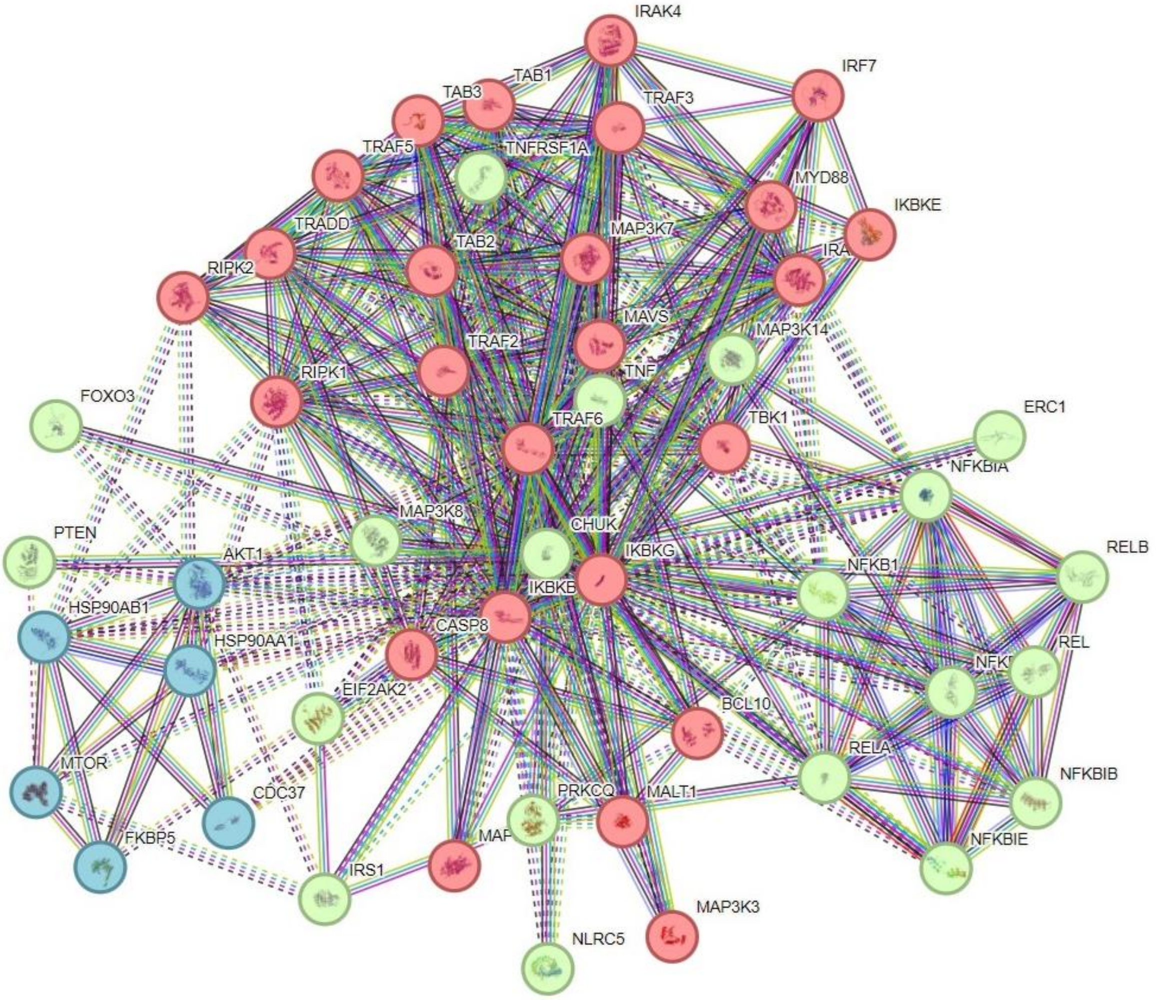

bubble	cluster Id	protein names
	Cluster 1	BCL10,CASP8,IKBKB,IKBKE,IKBKG,IRAK1,IRAK4,IRF7,MALT1,MAP3K1,MAP3K3,MAP3K7,MAVS,MYD88,RIPK1,RIPK2,TAB1,TAB2,TAB3,TBK1,TRADD, TRAF2,TRAF3,TRAF5,TRAF6
	Cluster 2	CHUK,EIF2AK2,ERC1,FOXO3,IRS1,MAP3K14,MAP3K8,NFKB1,NFKB2,NFKBIA,NFKBIB,NFKBIE,NLRC5,PRKCQ,PTEN,REL,RELA,RELB,TNF,TNFRSF1A
	Cluster 3	AKT1,CDC37,FKBP5,HSP90AA1,HSP90AB1,MTOR
