# Supplementary material for: Bioinformatic Analysis of IKK Complex Genes Expression in Selected Gastrointestinal Cancers
Source: Int J Mol Sci. 2024 Sep 12;25(18):9868. doi: 10.3390/ijms25189868 (PMC11432643; doi:10.3390/ijms25189868)
Supplement: Supplementary file 1 [file ijms-25-09868-s001.zip › Supplementary materials - Figure S23.pdf]

Supplementary materials - Figure S23. The cluster analysis for IKBKB by using STRING database (access 15-18.03.2024). The identified clusters are marked as red, green and blue.

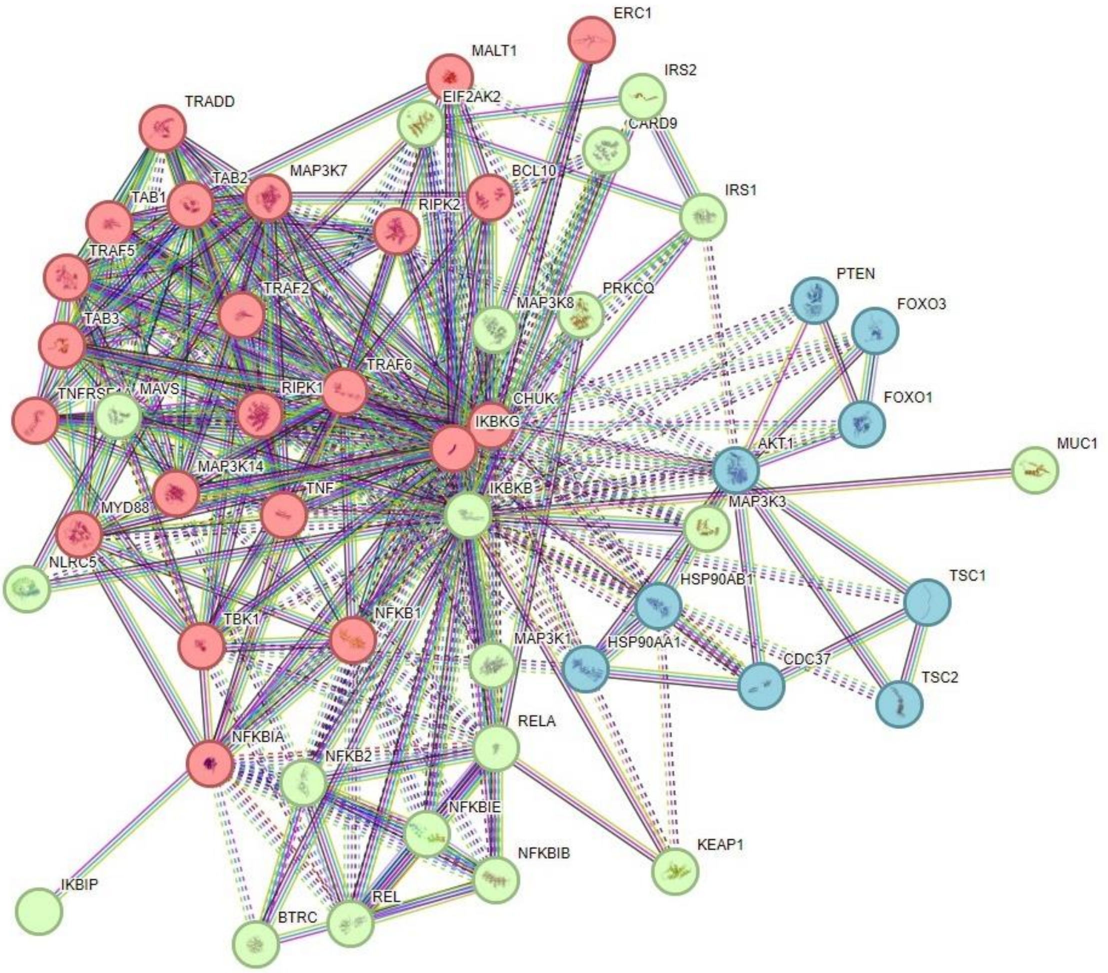

bubble	cluster Id	protein names
	Cluster 1	BCL10,CHUK,ERC1,IKBKG,MALT1,MAP3K14,MAP3K7,MYD88,NFKB1,NFKBIA,RIPK1,RIPK2,TAB1,TAB2,TAB3,TBK1,TNF,TNFRSF1A,TRADD,TRAF2, TRAF 5,TRAF6
	Cluster 2	BTRC,CARD9,EIF2AK2,IKBIP,IKBKB,IRS1,IRS2,KEAP1,MAP3K1,MAP3K3,MAP3K8,MAVS,MUC1,NFKB2,NFKBIB,NFKBIE,NLRC5,PRKCQ,REL,RELA
	Cluster 3	AKT1,CDC37,FOXO1,FOXO3,HSP90AA1,HSP90AB1,PTEN,TSC1,TSC2
