# Supplementary material for: Bioinformatic Analysis of IKK Complex Genes Expression in Selected Gastrointestinal Cancers
Source: Int J Mol Sci. 2024 Sep 12;25(18):9868. doi: 10.3390/ijms25189868 (PMC11432643; doi:10.3390/ijms25189868)
Supplement: Supplementary file 1 [file ijms-25-09868-s001.zip › Supplementary materials - Figure S24.pdf]

Supplementary materials - Figure S24. The cluster analysis for IKBKG by using STRING database (access 15-18.03.2024). The identified clusters are marked as red, green and blue.

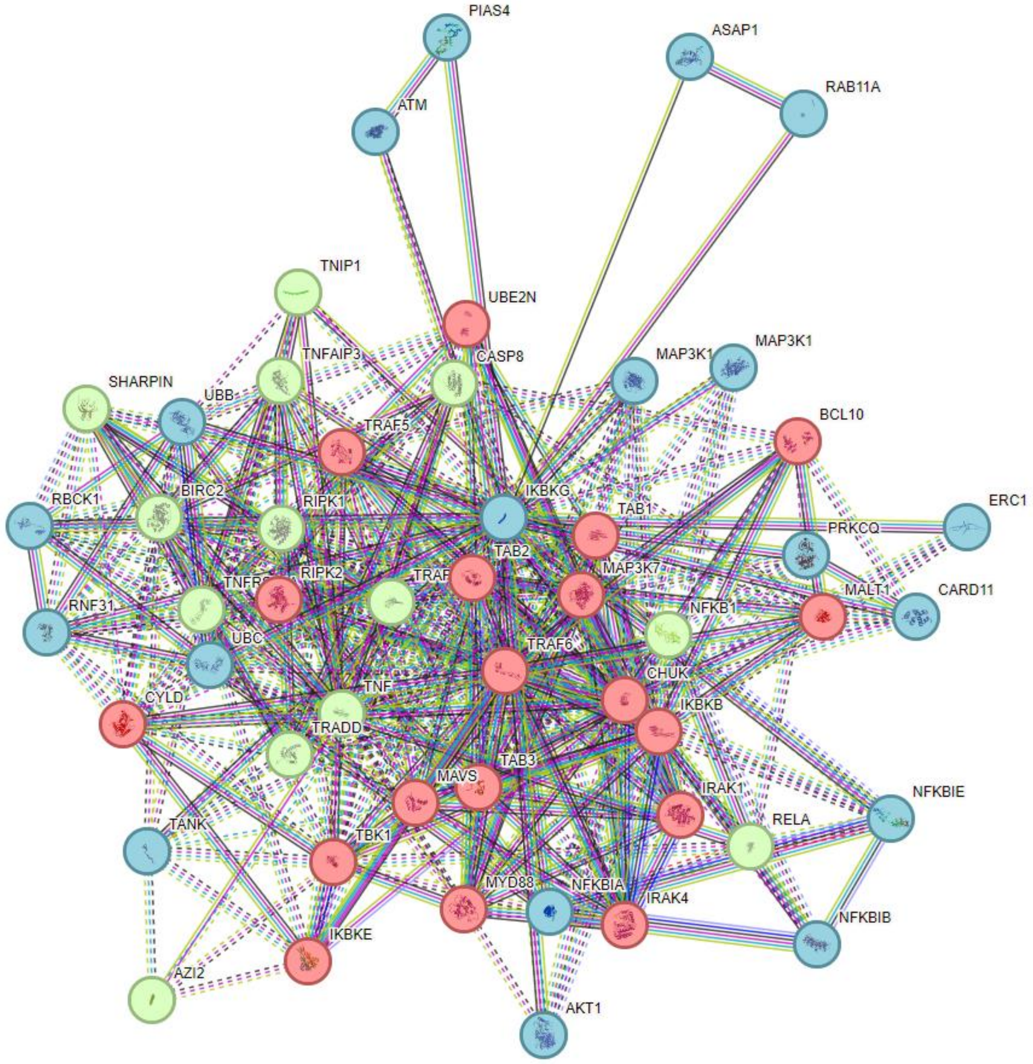

bubble	cluster Id	protein names
●	Cluster 1	BCL10,CHUK,CYLD,IKBKB,IKBKE,IRAK1,IRAK4,MALT1,MAP3K7,MAVS,MYD88,RIPK2,TAB1,TAB2,TAB3,TBK1,TRAF5,TRAF6,UBE2N
●	Cluster 2	AZI2,BIRC2,CASP8,NFKB1,RELA,RIPK1,SHARPIN,TNF,TNFAIP3,TNFRSF1A,TNIP1,TRADD,TRAF2
●	Cluster 3	AKT1,ASAP1,ATM,CARD11,ERC1,IKBKG,MAP3K1,MAP3K14,NFKBIA,NFKBIB,NFKBIE,PIAS4,PRKCQ,RAB11A,RBCK1,RNF31,TANK,UBB,UBC
