# Supplementary material for: Bioinformatic Analysis of IKK Complex Genes Expression in Selected Gastrointestinal Cancers
Source: Int J Mol Sci. 2024 Sep 12;25(18):9868. doi: 10.3390/ijms25189868 (PMC11432643; doi:10.3390/ijms25189868)

Supplementary materials - Figure S7. The correlation between somatic copy number variation in IKBKB gene and inflammatory response in COAD, ESCA,READ, STAD. The p-values are marked with asterisks: *: p < 0.05; **: p < 0.01; ***: p < 0.001 (access: 06-08.02.2023).

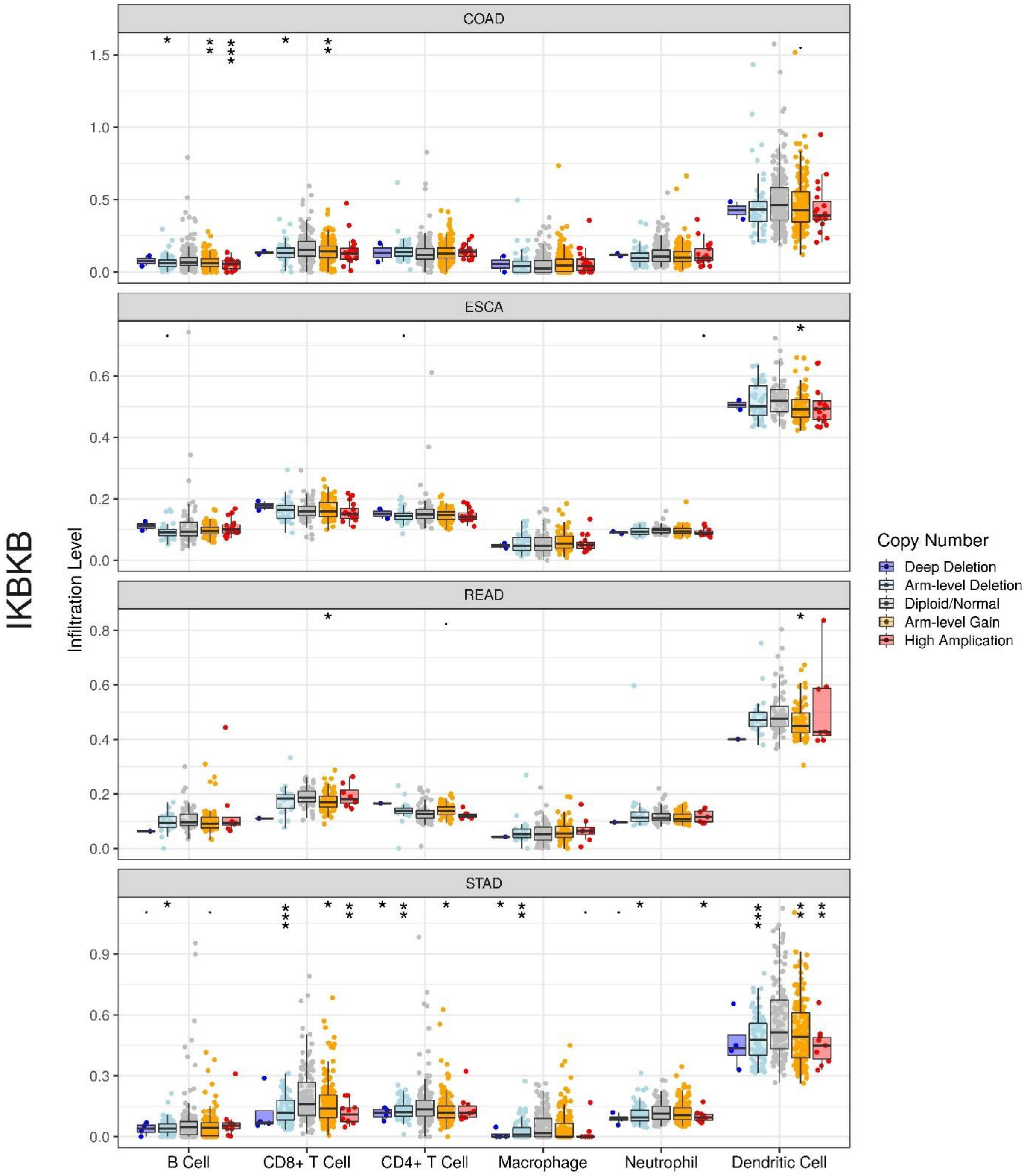

Supplement: Supplementary file 1 [file ijms-25-09868-s001.zip › Supplementary materials - Figure S7.pdf]
