# Supplementary material for: Bioinformatic Analysis of IKK Complex Genes Expression in Selected Gastrointestinal Cancers
Source: Int J Mol Sci. 2024 Sep 12;25(18):9868. doi: 10.3390/ijms25189868 (PMC11432643; doi:10.3390/ijms25189868)
Supplement: Supplementary file 1 [file ijms-25-09868-s001.zip › Supplementary materials - Table S1.pdf]

Supplementary materials - Table S1. The promoter methylation of IKK complex genes in selected GI cancers according to: individual clinical cancer stage, histological grade and nodal metastasis based on UALCAN web tool (Access: 04.09.2023).

		**COAD**			**ESCA**			**READ**			**STAD**	
	CHUK	***IKBKB***	***IKBKG***	***CHUK***	***IKBKB***	***IKBKG***	***CHUK***	***IKBKB***	***IKBKG***	***CHUK***	***IKBKB***	***IKBKG***
**Individual cancer stages**	***p***	***p***	***p***	***p***	***p***	***p***	***p***	***p***	***p***	***p***	***p***	***p***
Stage1-vs-Stage2	0,2123	0,7878	0,5848	0,2386	**0,0000**	0,1261	0,7741	0,4192	0,5285	0,9497	0,1995	0,1603
Stage1-vs-Stage3	0,3685	0,2044	0,9981	0,4026	**0,0021**	0,0521	0,1832	0,4890	0,4584	0,7369	0,0651	0,4884
Stage1-vs-Stage4	**0,0303**	0,5018	0,3708	0,6844	0,1164	0,0503	0,2376	0,8035	0,8478	0,6209	0,1287	0,8942
Stage2-vs-Stage3	0,6219	**0,0140**	0,5212	0,4512	0,3265	0,2615	0,3127	0,8973	0,7993	0,5898	0,3906	0,2867
Stage2-vs-Stage4	0,1387	0,1914	0,1273	0,4533	0,7140	0,4921	0,2913	0,2821	0,6763	0,5397	0,4813	0,2980
Stage3-vs-Stage4	0,0819	0,3949	0,3464	0,7777	0,9063	0,7800	0,1591	0,4483	0,5723	0,7221	0,8689	0,6717
**Tumor grade**												
Grade1-vs-Grade2	N/A	N/A	N/A	0,0829	0,3480	0,3623	N/A	N/A	N/A	**0,0215**	0,9821	0,3976
Grade1-vs-Grade3	N/A	N/A	N/A	**0,0171**	0,5221	0,3984	N/A	N/A	N/A	0,7494	0,2911	0,8439
Grade2-vs-Grade3	N/A	N/A	N/A	0,2400	0,7351	0,9007	N/A	N/A	N/A	**0,0025**	**0,0000**	**0,0015**
**Nodal metastatis status**												
N0-vs-N1	0,6447	**0,0105**	0,8282	0,1803	0,7651	0,0521	0,5154	0,5407	0,9489	0,0705	0,3210	0,1633
N0-vs-N2	0,4326	0,5202	0,9073	0,6061	**0,0413**	0,5844	0,2360	0,5851	0,9847	0,0503	0,6726	0,3781
N0-vs-N3	N/A	N/A	N/A	0,7952	0,8249	0,6464	N/A	N/A	N/A	0,9978	0,1340	0,5407
N1-vs-N2	0,3014	0,0994	0,9536	0,8159	**0,0274**	0,2199	0,1766	0,3722	0,9710	0,6911	0,6068	0,7011
N1-vs-N3	N/A	N/A	N/A	0,7286	0,9385	0,6515	N/A	N/A	N/A	0,0804	0,6713	0,5060
N2-vs-N3	N/A	N/A	N/A	0,8957	0,1084	0,3771	N/A	N/A	N/A	0,0560	0,3423	0,7994
